# High-Sensitivity Magnetic Particle Spectroscopy Platform for Precise, Specific, and Rapid Detection of Infectious Disease and Cancer-Related Biomarkers

**DOI:** 10.1021/acsmeasuresciau.6c00032

**Published:** 2026-06-29

**Authors:** Hafiz Ashfaq Ahmad, Ali Dinari, Minh Phu Bui, Seungjun Oh, Masoud Rezaie, Shahla Kianamiri, Jungwon Yoon

**Affiliations:** † Department of AI Convergence, 65419 Gwangju Institute of Science and Technology, Gwangju 61005, Republic of Korea; ‡ Department of Medical Physics, M. Smoluchowski Institute of Physics, 201871 Jagiellonian University, ul. S. Łojasiewicza 11, 30-348 Krakow, Poland; § Department of Chemistry, Jagiellonian University, Gronostajowa 2, 30-387 Cracow, Poland; ∥ Department of Nanotechnology, Agricultural Biotechnology Research Institute of Iran (ABRII), Agricultural Research, Education and Extension Organization (AREEO), 31359-33151 Karaj, Iran

**Keywords:** Magnetic particle spectroscopy, disease-related biomarkers, Aptamer-based biosensing, Brownian relaxation, magnetic nanoparticles conjugation

## Abstract

Accurate biosensing using magnetic particle spectroscopy (MPS) depends on the interplay between biomolecular binding and the fidelity of the measurement system. MPS enables wash-free, label-free biosensing by monitoring Brownian relaxation-induced changes in magnetic nanoparticle (MNP) dynamics. However, its practical performance is often limited by a weak signal-to-noise ratio, excitation feedthrough, environmental noise, slow acquisition, and insufficient mass sensitivity. Here, a gradiometer-based MPS system incorporating noise suppression, signal integration, and harmonic detection was developed to monitor relaxation changes upon biomolecular binding. Single-core SHP-25 MNPs were conjugated with recombinant human angiotensin-converting enzyme 2 (ACE2) protein, antinucleoprotein (anti_NP) antibodies, and AS1411 aptamer to target the SARS-CoV-2 receptor-binding domain (RBD), influenza A hemagglutinin type-1, neuraminidase type-1 (H1N1) nucleoprotein, and breast cancer biomarker nucleolin, respectively. Conjugation was validated by FT-IR spectroscopy and DLS to confirm chemical attachment and hydrodynamic size modulation, while ICP-MS was used for iron-mass normalization. Relaxation changes from conjugation and target binding were recorded by MPS. The third-harmonic showed the highest sensitivity to binding-induced relaxation delays, with detection limits of 1 nM (100 fmol) for ACE2 and anti_NP and 12 nM for AS1411. Target-binding yielded statistically significant responses at concentrations as low as 5 nM for RBD, H1N1, and nucleolin. The harmonic ratio R_53_ (fifth/third) increased monotonically with binding, providing a mass-normalized recognition metric with reduced dependence on nanoparticle concentration. The system achieved an iron-mass sensitivity of 1 ng Fe. Comparative analysis shows that monoclonal antibody-conjugated MNPs provide greater specificity and quantitative robustness in MPS measurements than polyclonal antibodies. The high sensitivity of the developed MPS, combined with the superior specificity of monoclonal antibodies, enables precise biosensing with potential clinical relevance.

## Graphical abstract

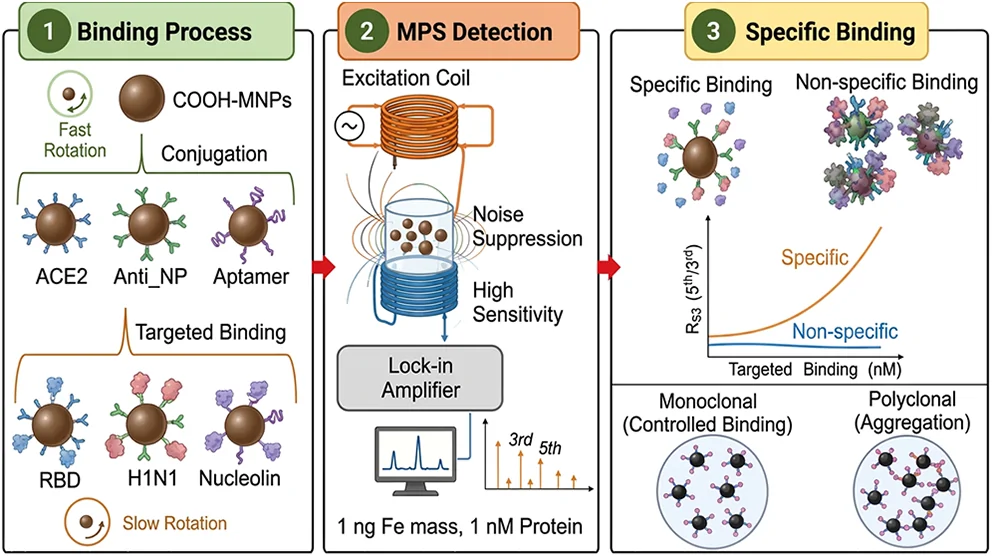

## Introduction

The accuracy of diagnosis acts as a milestone in disease therapy. Accurate assessment of the disease state is essential for selecting the appropriate therapeutic strategy. Literature review with diagnostic systems shows the enormous research that leads to finding the state-of-the-art diagnostic modalities.^1–4^ Numerous biosensing platforms based on electrical, electrochemical, acoustic, magnetic, colorimetric, and fluorescent transduction mechanisms have been reported.^5^ Detection strategies aim to improve sensitivity, expand the range of detectable agents, and reduce detection time.^6^ Recent advances have emphasized integrating modality sensitivity with molecular specificity, enabling accurate, noninvasive detection of disease biomarkers at early stages. Among these modalities, optical and magnetic techniques have shown considerable promise.^7,8^

Optical diagnostic methods like ultraviolet (UV), visible light, near-infrared (NIR) fluorescence, surface plasmon resonance (SPR), and surface-enhanced Raman scattering (SERS) show promising results but are limited by tissue interference, shallow penetration depth, and susceptibility to background interference.^9,10^ On the other hand, tomographic modalities, such as computed tomography (CT), positron emission tomography (PET), and magnetic resonance imaging (MRI), provide noninvasive clinical imaging, but are typically costly, time-consuming, and may involve radiation exposure or indirect signal sources.^11–13^ In contrast, magnetic particle imaging (MPI) offers a key advantage: its signals originate solely from magnetic tracers, with no background interference from tissues. MPI is noninvasive, radiation-free, and directly measures the magnetization of MNPs, providing high specificity and quantitative accuracy.^14–18^ Magnetic particle spectroscopy (MPS), a zero-dimensional form of MPI, measures the dynamic magnetic response of MNPs independent of spatial location.^19^

MPS probes the nonlinear magnetic response of MNPs under alternating magnetic fields,^20^ governed by two relaxation mechanisms: Néel (magnetization flipping within particles) and Brownian (physical rotation of particles).^21^ The dominant relaxation mechanism depends on the magnetic core sizes of the MNPs^22^ as well as particle and environmental properties. For single-core iron oxide nanoparticles, Brownian relaxation is often more prominent above approximately 20 nm, although this is not a strict cutoff.^23^ This process reflects the degree of freedom in the physical rotational motion of MNPs. Any surface attachment, such as proteins or nucleic acids, increases the hydrodynamic diameter and alters Brownian motion, resulting in measurable changes in the MPS signals.^24,25^ The first successful application of MPS was demonstrated by Cooley,^26^ with a sensitivity of only 45 ng, making it challenging to use for biological applications. Subsequently, the designed MPS system, having both a gradiometer coil and a calibration coil, achieved a detection limit of 25 ng.^19^ However, the addition of a calibration coil increased receive-channel impedance and introduced phase imbalance, ultimately limiting signal cancellation efficiency and sensitivity. More recently, MPS has emerged as a promising platform for real-time, wash-free biosensing and has been applied to viral biomarker detection, including influenza antigens and SARS-CoV-2–related targets.^20,25,27–29^

Conventional MPS-based biosensing remains sensitive to nanoparticle concentration because harmonic amplitudes depend on both particle mobility and total magnetic content. Recently, critical-offset approaches such as Critical Offset Magnetic PArticle SpectroScopy (COMPASS) have been introduced to improve robustness in signal interpretation by applying an additional DC offset magnetic field and probing mobility-sensitive critical points in the harmonic response.^30^ However, this approach involves added measurement complexity and still needs broader validation across different assay systems. Moreover, many prior MPS-based viral assays rely on polyclonal antibodies, which may introduce inherent limitations: polyclonal binding heterogeneity can potentially promote particle–particle cross-linking, allow multiple MNPs to interact with the same biomarker molecule, broaden the distribution of binding orientations, and increase the likelihood of nonspecific nanoparticle aggregation.^24,28,29^ Such aggregation, if present, can independently alter hydrodynamic size and Brownian mobility, making it difficult to isolate true biomolecular binding effects induced by antibody-mediated clustering. Therefore, improved MPS biosensing strategies that minimize aggregation-induced artifacts and enable controlled, molecule-specific detection remain needed.

In this study, a highly sensitive MPS system was developed incorporating a gradiometric coil design to minimize excitation feedthrough and environmental interference. Optimized coil design, precise phase and amplitude compensation, combined with frame integration, substantially enhanced the signal-to-noise ratio, achieving an iron-mass sensitivity of 1 ng Fe within 20 s, enabling accurate biomolecular interaction analysis. For specific detection of viral and cancer-associated biomarkers, MNPs were conjugated with well-defined biorecognition elements. Recombinant human angiotensin-converting enzyme 2 (ACE2) monoclonal serves as the native binding partner of the SARS-CoV-2 spike receptor-binding domain (RBD), monoclonal antinucleoprotein (anti_NP) antibodies were used for selective recognition of influenza A (H1N1) nucleoprotein, and the AS1411 aptamer, a 26-base G-rich DNA sequence with strong affinity for nucleolin, was selected to target a breast-cancer-related biomarker.^31,32^ These monoclonal antibodies, proteins, and aptamers were covalently attached to carboxyl-functionalized MNPs via EDC/NHS coupling, ensuring molecular specificity and controlled biorecognition during subsequent MPS measurements.

The sensitivity of the developed MPS device was validated using Synomag and SHP-25 nanoparticles. Synomag particles were chosen for their mixed Brownian-Néel relaxation behavior in the midfrequency (10–30 kHz) range, whereas SHP-25 particles exhibit predominantly Brownian relaxation and provide stronger size-dependent responses at lower frequencies. MPS measurements were performed at 24.36 kHz for Synomag and 7.74 kHz for SHP-25, using the third harmonic amplitude as the primary analytical metric.^28,33^ After instrument validation, SHP-25 MNPs were conjugated with the selected biorecognition elements via EDC-NHS coupling. Conjugation was confirmed by Fourier-transform infrared spectroscopy (FT-IR) and dynamic light scattering (DLS), and inductively coupled plasma mass spectrometry (ICP-MS) was used to quantify iron content in plain and conjugated MNPs.^25^ All samples were normalized to identical iron masses to ensure that MPS signal variations arose solely from binding-induced relaxation changes. This two-step scheme, consisting of primary probe conjugation followed by secondary target recognition, enabled nanomolar-level detection of protein- and aptamer-based interactions. Comparative analysis further reveals that monoclonal antibodies-functionalized MNPs provide superior specificity, reproducibility, and quantitative robustness in MPS measurements compared to polyclonal antibodies, underscoring their suitability for precise biomarker detection.

## Materials and Methods

### Materials

Recombinant human angiotensin-converting enzyme 2 (ACE2, hFc tag; Cat. No. 10108-H02H), SARS-CoV-2 (2019-nCoV) spike receptor-binding domain (RBD, His tag; Cat. No. 40592-V08H), influenza A H1N1 (A/Puerto Rico/8/34/Mount Sinai) nucleoprotein (NP, I116M, ECD, His tag; Cat. No. 11675-V08B), were purchased from Sino Biological Inc. (China), and Human Nucleolin/NCL Protein (fc tag; Cat. No. NUL-H5253) was purchased from ACRO-biosystems (Switzerland). Polyclonal spike RBD antibody (rabbit IgG; Cat. No. 40592-T62) and polyclonal influenza A nucleoprotein antibody (rabbit IgG, antigen affinity purified; Cat. No. 11675-T62) were also obtained from Sino Biological Inc. (China). Synomag magnetic nanoparticles (5 mg/mL; Cat. No. 125–01–501) were purchased from Micromod Partikeltechnologie GmbH (Rostock, Germany), and carboxyl-functionalized iron oxide nanoparticles (SHP25–02; Lot No. 040721) were obtained from Ocean NanoTech Inc. (USA). MES buffer (2-(N-morpholino) ethanesulfonic acid hydrate; CAS No. 4432–31–9), 1-ethyl-3-(3-(dimethylamino)­propyl) carbodiimide (EDC), and *N*-hydroxysuccinimide (NHS) were purchased from Sigma-Aldrich (South Korea). Bis­(sulfosuccinimidyl) suberate (BS3), phosphate-buffered saline (PBS; Cat. No. 10010031), and deionized (DI) water were obtained from Thermo Fisher Scientific (South Korea). The AS1411 aptamer (26-base guanine-rich oligodeoxynucleotide) was purchased from Bio-Synthesis Inc. (USA). Dialysis tubing (SnakeSkin Dialysis, 3.5 kDa MWCO; Cat. No. 88242) was obtained from Thermo Fisher Scientific (South Korea).

### Magnetic Particle Spectroscopy (MPS)

#### Design

A MPS system was developed, containing excitation coil for magnetic field generation and the receiver coil for signal acquisition from MNPs. The excitation coil was constructed by winding five layers (5 × 75 turns) of Litz wire (140/46) around a 3D-printed cylinder (100 mm length, 20 mm diameter) to generate a uniform alternating magnetic field. Litz wire was selected to minimize eddy current losses at high frequency. An AC excitation signal was generated using a function generator (Keysight 33500B series) and amplified with a linear power amplifier (Model 7224, AE Techron, Elkhart, IN, USA). A band-pass filter was employed to suppress harmonic distortion and ensure single-frequency excitation.^34^ To achieve high sensitivity, the receiver coil was designed following the optimal geometric ratio length *l*/*D* = 0.866, where *l* is the length and *D* is the diameter.^35^ A first-order gradiometer configuration was employed, comprising a receive coil and a cancellation coil with identical geometry (24 mm length, 12 mm diameter, 88 turns, 10/46 Litz wire) but wound in opposite directions to nullify the primary excitation field. This configuration minimized feedthrough and improved the signal-to-noise ratio by ensuring near-zero output in the absence of MNPs.^36^ Due to the nonlinear magnetization of MNPs, higher-order harmonic signals were induced in the receive channel, which is further processed through a receive filter (Model SR650) and a Lock-In amplifier (SR865A 4 MHz DSP Lock-in Amplifier) to measure the third and fifth harmonics of the sample’s signal. Higher harmonics were used instead of the fundamental frequency to avoid picking up the signal of the applied field. The odd harmonics are of interest in describing the shape of magnetization and its saturation.^37^ The ratio of the fifth to third harmonic amplitudes (R_53_) provides a partially normalized metric that is less sensitive to nanoparticle concentration than absolute harmonic amplitudes and remains sensitive to Brownian relaxation behavior.^38^ The processed signal is digitized and recorded using data acquisition modules (NI PXIe-7867 and PCIe-6363, National Instruments, Austin, TX, USA). Amplification and filtering stages were tuned and frame-integration method was used to suppress environmental and electronic noise. To minimize temperature-related drift, the MPS system was operated at a low excitation field (2 mT) and measured using an on/off duty cycle. The ambient temperature around the setup was maintained at or slightly below room temperature, and all measurements were conducted under consistent laboratory conditions and timing to reduce temperature-induced variability. The developed MPS system is shown in Figure 1A,B.

**Figure 1:**
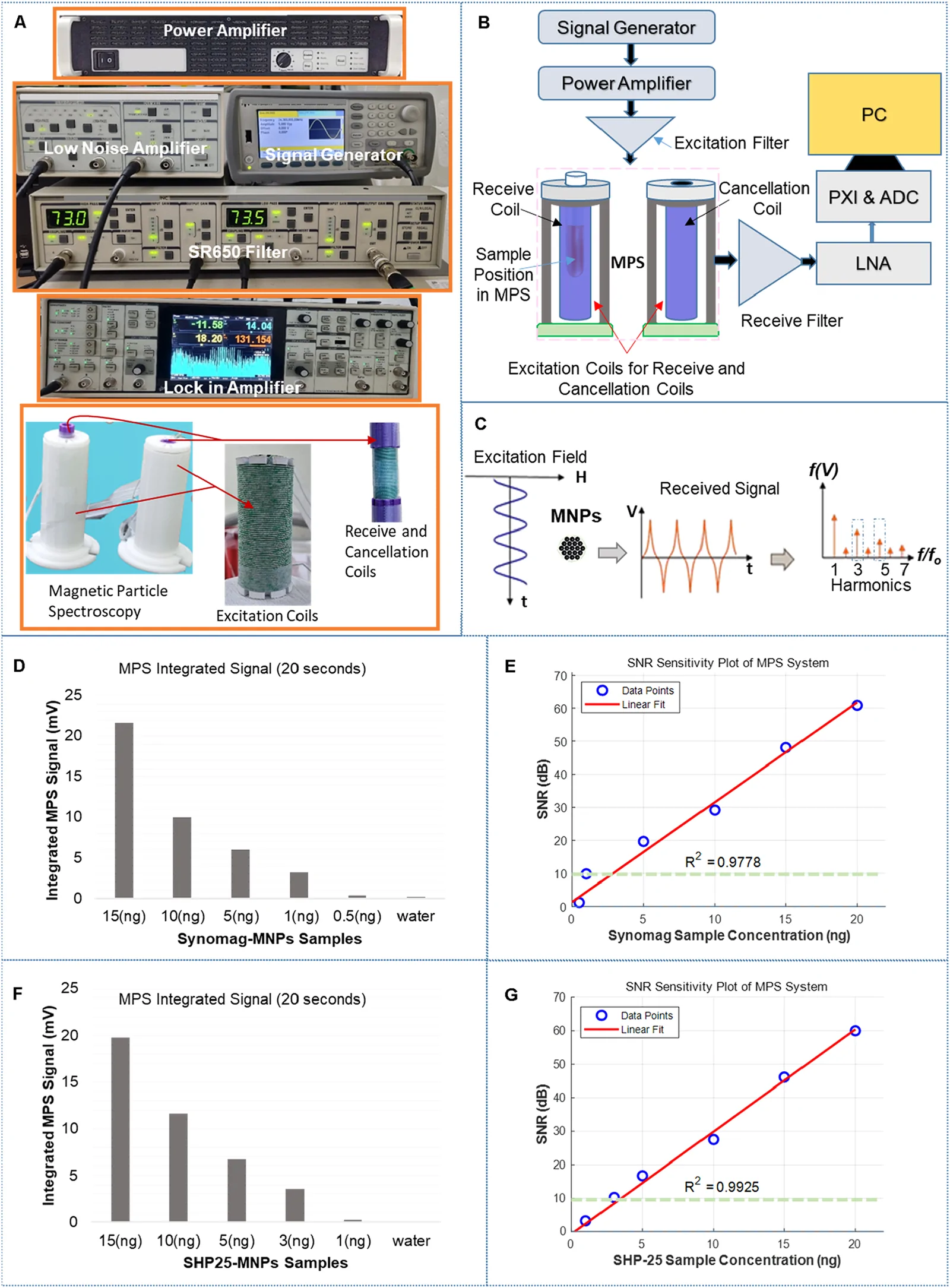
Overview of the developed MPS system. (A) shows the physical hardware implementation of the system, highlighting the signal generator, power amplifier, lock-in amplifier, and other components. (B) presents the schematic layout of the MPS system. The system comprises two main parts: the excitation circuit and the receive circuit. The excitation circuit consists of a signal generator, power amplifier, excitation filter, and two excitation coils connected in series to generate a strong and uniform alternating magnetic field. The receive circuit includes a receive coil, cancellation coil, receive filter, Lock-In amplifier, PXI-based analog-to-digital converter (ADC), and a computer for signal acquisition and processing. (C) illustrates the working principle of MPS. Under the influence of an external oscillating magnetic field, MNPs induced a nonlinear response. The system detects the time-domain signal, which is then transformed into the frequency domain for harmonic analysis. Results parts shows the sensitivity assessment of the MPS using Synomag and SHP-25 nanoparticles. (D) Third-harmonic RMS response of Synomag samples illustrating a prominent signal at 1 ng. (E) Corresponding SNR analysis (dB scale), demonstrating a detection limit of 1 ng Fe. (F) Third-harmonic RMS response of SHP-25 samples under identical acquisition conditions. (G) SNR-derived sensitivity plot indicating a detection threshold of 3 ng Fe. Distilled water was used as the negative control to verify magnetic signal specificity.

#### Calibration of the MPS System

The system was calibrated to minimize residual excitation-field coupling in the gradiometer configuration. Under experimental conditions, the magnetic field inside the excitation coil is not perfectly uniform. In our gradiometer coil, the residual voltages are defined as in (1)^39^

1 $$V_{\mathrm{residual}}=\sqrt{V_{\mathrm{rec}}^{2}+V_{\mathrm{can}}^{2}+2V_{\mathrm{rec}}V_{\mathrm{can}}\cos\alpha }$$

Where *V*_rec_ and *V*_can_ are the voltage amplitudes of the receive coil and cancellation coil, respectively, and α is the phase difference between the two signals from the receive coil and the cancellation coil. To attenuate the residual voltage to zero, the phase should be equal to π. The phase difference is given by

2 $$\alpha =\arctan\left(\frac{\omega L_{\mathrm{rec}}}{R_{\mathrm{rec}}}\right)-\arctan\left(\frac{\omega L_{\mathrm{can}}}{R_{\mathrm{can}}}\right)-\pi$$

The phase difference α depends on the inductors (*L*_rec_, and *L*_can_) and the resistors (*R*_rec_, and *R*_can_) of the receive and cancellation coils, respectively. Using a LCR meter (B&K Precision Model 894) to measure the inductance and resistance of each coil, the phase difference α can be adjusted through adjusting the turns of wire of the cancellation coil, effectively reducing residual voltage to approximately 2 mV with an attenuation of 44 dB. This calibration step ensured consistent and reproducible measurements.

#### MPS Sensitivity Test

To evaluate system sensitivity, Synomag MNP samples containing 15, 10, 5, 1, and 0.5 ng of iron in 100 μL were prepared, along with SHP-25 MNP samples containing 15, 10, 5, 3, and 1 ng of iron in 100 μL. Measurements were performed using the third harmonic component to optimize analog-to-digital conversion performance. When multiple harmonics are present in the output signal, a significant white Gaussian noise is added to the signal.^40^ To mitigate additive white Gaussian noise, signal integration over multiple frames was employed to further reduce noise contributions.^19^ Assuming that the output signal *U*_signal_(t) of the gradiometer coil measured in one-time frame (0.5 ms) includes the MNPs signal *U*_p_(t) and the white Gaussian noise signal *U*_noise_(t)

3 $$U_{\mathrm{signal}}\left(t\right)=U_{p}\left(t\right)+U_{\mathrm{noise}}\left(t\right)$$

The integrated signal *IntSig*(*t*) was obtained by summing the output signal during *n* frames.

4 $$\mathrm{IntSig}\left(t\right)=\sum_{\mathrm{frame}=1}^{n}U_{\mathrm{signal}}\left(t\right)=n\times U_{p}\left(t\right)+\sum_{1}^{n}U_{\mathrm{noise}}\left(t\right)$$

In the integrated signal, as *n* increases, the total measurement time becomes long enough that the white Gaussian noise component averages out and reduced with increasing frame integration, but is not eliminated entirely: ∑_1_^*n*^ *U*_noise_(*t*) → 0. Thus, the root-mean-square (RMS) value of *IntSig*(*t*) represents the final output signal induced by MNPs. Here, one frame corresponds to a 0.5 ms acquisition window, and frame integration was performed by repeatedly acquiring these short frames and numerically summing them over n frames. The RMS value of the integrated signal was then used as the reported output. This approach improved detectability under low-signal conditions by reducing the effective noise floor.

### Dialysis Process

The recombinant proteins (such as monoclonal RBD, ACE2, H1N1 and anti_NP) were supplied in powder form with a buffer containing Tris and glycine, which can interfere with EDC/NHS coupling chemistry. To eliminate buffer-related interference, all protein samples were subjected to dialysis prior to conjugation. Detailed dialysis procedures are in Supporting Information Scheme S1.

### Conjugation Process

Conjugation of biomolecules to carboxyl-functionalized iron oxide nanoparticles (COOH-MNPs) was performed using the EDC/NHS coupling method.^41^ Briefly, COOH-MNPs were activated in 100 mM MES buffer (pH 6.5) containing 10 mM EDC and 5 mM NHS and incubated for 10 min. Subsequently, the activated nanoparticles reacted with proteins or the AS1411 aptamer.^33^ The AS1411 aptamer was premodified with a terminal amine group to facilitate covalent attachment. Following conjugation, unbound biomolecules were removed by washing the nanoparticles three times with PBS.

### FT-IR Spectroscopy

FT-IR spectroscopy identifies the chemical makeup of substances by analyzing their infrared light absorption patterns.^42^ FT-IR spectra of unmodified MNPs and MNPs conjugated with ACE2, anti_NP, and AS1411 aptamer at concentration 30 nM were acquired using an FT-IR spectrometer (Frontier, PerkinElmer, USA) after mixing samples with KBr powder. The spectra were collected over the range of 4000 – 400 cm^–1^, with a spectral resolution set to 0.4 cm^–1^ and wavenumber precision down to 0.008 cm^–1^. Characteristic amide I and amide II absorption bands were analyzed to confirm covalent coupling between the protein and the MNP surface.^33^ Sample details are listed in Supporting Table S1.

### DLS Measurements

The hydrodynamic size and zeta potential of protein- and aptamer-conjugated MNPs were characterized using dynamic light scattering (Zetasizer Nano ZSP, Malvern Panalytical, UK). Conjugated nanoparticle dispersions were prepared at appropriate concentrations ranging from 50 to 100 μg/mL. In all samples, the concentration of MNPs was fixed at 5 μg. Biomolecule conjugation was performed using 15 nM, and 30 nM of ACE2, anti_NP, and AS1411 aptamer. Samples details are shown in Supporting Table S1. DLS measurements were performed at room temperature, and data were recorded for both hydrodynamic diameter (*Z*-average) and zeta potential.^33^

### Inductively Coupled Plasma Mass Spectrometer (ICP) Analysis

To ensure that MPS readouts reflected Brownian-relaxation changes rather than variations in magnetic loading, the iron content of all samples (plain and conjugated MNPs) was quantified by ICP-MS (Agilent 7900, Japan) following microwave-assisted acid digestion (Multiwave 7000, Anton Paar) and then equalized across samples.^25^ Conjugated suspensions were magnetically separated, washed, and resuspended in 200 μL PBS. A volume of 10 μL from each sample was transferred to a digestion vessel, combined with 1,000 μL nitric acid, and digested using the instrument protocol.^25^ After cooling, the digests were diluted to the working volume with pure water and put in ICP-MS vials for analysis. Iron concentration was determined using external calibration with Fe standards and procedural blanks, and the measured values were then used to normalize all samples to an identical iron mass prior to MPS measurements.

### MPS Measurements of Conjugated MNPs

MPS measurements were performed to evaluate the capability of the developed system to detect binding-induced changes in MNPs relaxation behavior. The experiments were conducted in three sequential steps to assess sensitivity to primary surface conjugation and secondary biomolecular recognition. In the first step, MNPs were conjugated with varying concentrations of ACE2, anti_NP antibodies, and AS1411 aptamer. For ACE2 and anti_NP, concentrations of 0.5, 1, 2, 4, 8, and 16 nM were tested, while AS1411 was evaluated at 6, 12, 18, 24, 30, and 36 nM. MPS measurements were performed after conjugation to assess binding-induced relaxation changes. In the second step, fixed concentrations of primary biomolecules (8 nM for ACE2 and anti_NP antibodies and 24 nM for the AS1411 aptamer) were first conjugated to the MNPs. Subsequently, target monoclonal proteins were introduced to form specific biomolecular complexes: SARS-CoV-2 RBD for ACE2-conjugated MNPs, influenza A (H1N1) nucleoprotein for anti_NP-conjugated MNPs, and nucleolin for AS1411-conjugated MNPs. Secondary binding was evaluated at target concentrations of 1, 5, 10, 15, and 20 nM. In the third step, polyclonal versions of the same target proteins were prepared at identical concentrations (1, 5, 10, 15, and 20 nM) to enable direct comparison with monoclonal target binding. Sample identifiers and experimental conditions are summarized in Supporting Tables S2 and S3, and the corresponding MPS responses are presented in the Results section. Additional nonspecific control experiments were also performed by incubating unconjugated COOH-functionalized SHP-25 MNPs directly with RBD, H1N1 nucleoprotein, or nucleolin over the same concentration range used in the targeted binding studies as described in Supporting Information Scheme S4.

## Results and Discussion

### MPS Sensitivity Test

The sensitivity of the developed MPS system was evaluated by using Synomag and SHP-25 nanoparticles (stock concentration: 5 mg/mL) serially diluted with distilled water to prepare test samples containing nanogram iron quantities (sample details provided in the Materials and Methods section). Distilled water without nanoparticles served as the negative control. Sensitivity measurements were conducted using a low-amplitude alternating magnetic field of 2 mT at excitation frequencies of 24.36 kHz, for Synomag and 7.74 kHz for SHP-25. The samples were placed one by one in the MPS, and the resulting harmonic signals were recorded. A clear distinction was observed between nanoparticle-containing samples and the control sample, indicating reliable detection of magnetic relaxation-derived signals even at low particle concentrations (Figure 1D–G). For Synomag, the signal difference between 15 ng of Fe and the control was resolvable within a single acquisition frame (0.5 ms) and within 0.5 s using frame integration (*n* = 1000), demonstrating that signal integration enhances detectability while preserving quantitative accuracy. The measured third-harmonic RMS amplitudes for 10 ng, 5 ng, and 1 ng of Fe (integrated over 2000, 10 000, and 40 000 frames, respectively) are presented in Figure 1D. For SHP-25, detectable third-harmonic signals were obtained at 10 ng, 5 ng, and 3 ng of Fe using the same integration scheme (Figure 1F). Sensitivity plots based on signal-to-noise ratio (SNR, expressed in dB) are shown in Figures 1E,1G. In our measurements, the overall trend remained close to linear, as supported by the linear fits of the SNR plots in Figure 1E,1G, which showed *R* ^2^ = 0.9778 for Synomag and *R* ^2^ = 0.9925 for SHP-25. These values indicate good linear behavior with only minor experimental deviation and demonstrate that the system achieves a detection limit of 1 ng Fe for Synomag nanoparticles and 3 ng Fe for SHP-25 nanoparticles. The selected excitation frequencies were based on particle-specific frequency-response measurements in the developed MPS system, which showed maximum third harmonic responses at 24.36 kHz for Synomag and 7.74 kHz for SHP-25 (see Supporting Information Figure S3 and Table S6).

Synomag particles are multicore composites with inherently restricted Brownian motion, making their magnetic response largely insensitive to clustering. Conversely, SHP-25 single-core particles retain full Brownian mobility, yielding higher sensitivity to hydrodynamic-size changes and improved performance in relaxation-based biosensing.^33,43^ For this reason, SHP-25 MNPs were employed for all subsequent biomarker detection measurements.

### FT-IR Spectral Analysis for Protein Conjugation Validation

FT-IR spectroscopy confirmed the conjugation of ACE2, anti_NP proteins, and AS1411 aptamer to carboxyl-functionalized MNPs (Figure 2A). The conjugation was carried out using EDC-NHS chemistry, which enables the formation of stable amide bonds between carboxyl (−COOH) and amine (−NH_2_) groups; this process is typically reflected in changes in the amide I (∼1650 cm^–1^, C=O stretching) and the amide II band (∼1540 cm^–1^, N–H bending and C–N stretching). All samples displayed a distinct Fe–O vibration at ∼580 cm^–1^, confirming the iron oxide core.^42^ Broad O–H/N–H stretching peaks between 3200 cm^–1^ and 3400 cm^–1^ were evident for both bare and conjugated MNPs, originating from surface hydroxyl groups and adsorbed water; however, this band became broader and more intense after conjugation due to additional N–H and O–H vibrations from attached biomolecules. In bare MNPs, additional spectral peaks were noted at 1340, 1502, 1657, and 1687 cm^–1^, which were assigned to carboxylate (COO^–^) surface vibrations, possible C–H bending modes, and reacted carbonyls. These peaks weakened and disappeared after surface modifications, as expected due to the use or transformation of surface carboxylate groups during the formation of amide bonding. The feature at about 1540 cm^–1^ strengthened after modification, as a result of new amide II features along with any remaining carboxylate signals. However, it should be noted that the absorption band around 1700–1730 cm^–1^ for free carboxylate C=O stretching, which exists in free form for all carboxylates and is largely deprotonated to COO^–^ on the surface, was not observed for either initial or modified particles. Indeed, all carboxylates would be expected to be largely consumed/reacted during modification. Therefore, the detailed changes in FT-IR spectra, including increased intensity at amide regions, increased intensity at 3200–3400 cm^–1^, and a decrease at 1340, 1502, 1657, and 1687 cm^–1^ regions, all verify successful surface biomolecule conjugation at a correct concentration.^44,45^

**Figure 2:**
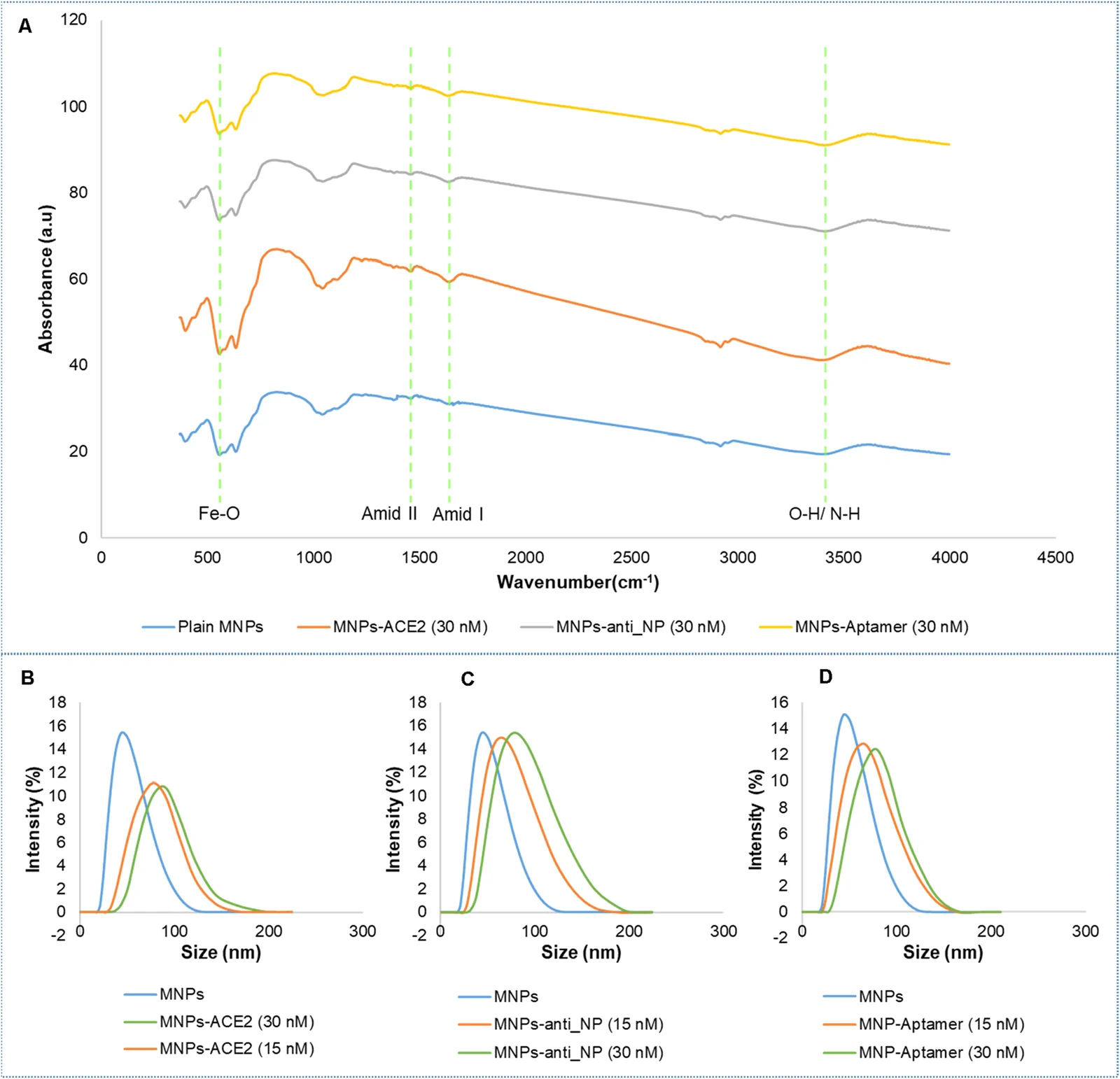
(A) FT-IR spectra of unmodified MNPs (bottom), MNPs conjugated with 30 nM of ACE2, anti_NP, and AS1411 aptamer are shown, respectively. The 3200–3400 cm^–1^ (O–H/N–H stretch) and 1650/1540 cm^–1^ (amide I/II) regions confirm successful surface attachment of biomolecules. (B–D) shows the hydrodynamic size distribution of MNPs after protein and aptamer conjugation, measured by DLS. (B) Size profiles of plain MNPs and increasing concentrations of ACE2-conjugated MNPs. (C) Size profiles of anti_NP-conjugated MNPs. (D) Size profiles of aptamer-conjugated MNPs. In all cases, conjugation resulted in a concentration-dependent increase in hydrodynamic diameter, confirming successful surface binding of biomolecules.

### DLS Results

Conjugation of biomolecules to the surfaces of MNPs is expected to increase their hydrodynamic size, which was evaluated using DLS analysis. Table 1 summarizes the hydrodynamic size of unmodified MNPs and MNPs conjugated with ACE2, anti_NP, and aptamer at varying concentrations (see Supporting Table S1 for sample details). The average hydrodynamic size of unmodified MNPs was 51 nm, which increased to 71.8 nm upon conjugation with ACE2 at a concentration of 30 nM, in the case of anti_NP-conjugated MNPs, the size increased to 84 nm, and for aptamer-conjugated MNPs the size shifted to 59.55 nm. These systematic size increases indicate successful conjugation via EDC-NHS chemistry. In addition to size changes, zeta potential and polydispersity index (PDI) analyses were used to assess surface charge modification and colloidal stability. The zeta potential of unmodified MNPs (−22.9 mV) became progressively less negative following ACE2 conjugation, reaching −12.33 mV at 30 nM, consistent with surface charge neutralization by covalently attached protein amine groups. Correspondingly, PDI increased from 0.117 to 0.262, reflecting increased heterogeneity due to protein layering. A similar trend was observed for anti_NP conjugation, with zeta potential shifting to −8.36 mV and PDI increasing to 0.30 at 30 nM, indicating a broader size distribution at higher protein loading. Aptamer-conjugated MNPs exhibited a concentration-dependent response, with zeta potential shifting to −15.26 mV at 15 nM and stabilizing at −16.36 mV at 30 nM, accompanied by PDI values remaining below 0.2. The DLS intensity distributions in Figure 2B–D show a clear rightward shift of the size peaks for all conjugated MNPs relative to unmodified particles, with larger shifts observed at higher biomolecule concentrations, consistent with increased surface coverage following conjugation. Notably, anti_NP-conjugated MNPs exhibit the most pronounced peak broadening, whereas aptamer-conjugated MNPs display a narrower shift, reflecting differences in biomolecule size and surface packing while maintaining unimodal distributions indicative of stable, nonaggregated suspensions. It should be noted that the DLS size increase reflects hydrodynamic diameter in solution, not the exact thickness of a molecular layer, and therefore does not by itself indicate multiple antibody layers.^46^ Overall, the combined DLS, zeta potential, and PDI results (Figure 2 and Table 1) confirm successful biomolecule conjugation while maintaining acceptable colloidal stability.

**Figure 3:**
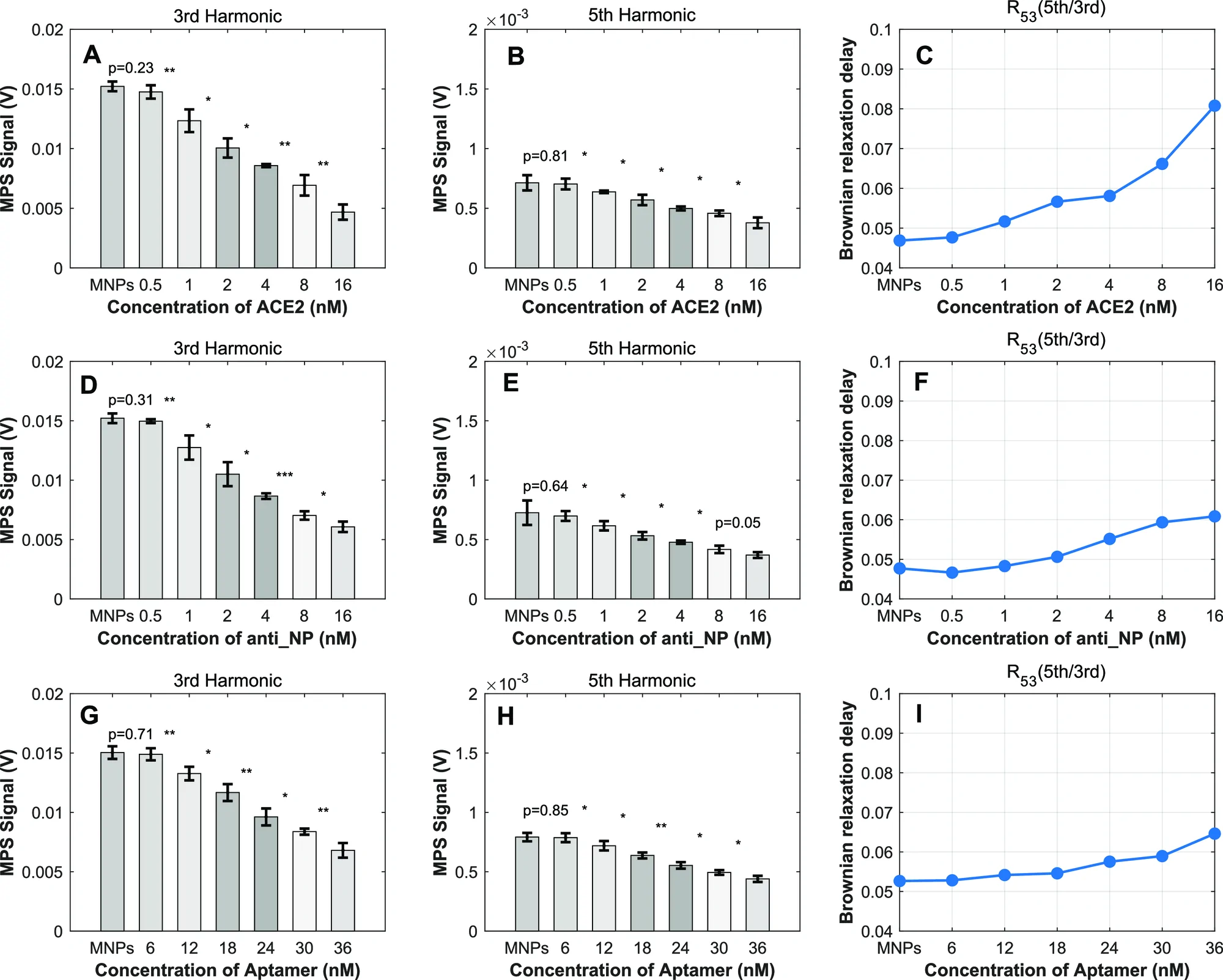
MPS response to primary conjugation of MNPs with ACE2, anti_NP, and AS1411 aptamer. (Left to right) third harmonic amplitudes, fifth harmonic amplitudes, and the corresponding harmonic ratio R_53_ = fifth/third. All systems show a concentration-dependent attenuation of the third harmonic, accompanied by a more modest decrease in the fifth harmonic. The ratio R_53_ increases monotonically with biomolecule loading, serving as a quantitative indicator of relaxation delay. Error bars show standard deviations from independent measurements.

**Table 1:** Hydrodynamic Size and Zeta Potential Values of Unmodified and Conjugated MNPs (Values Averaged across All Samples at Each Biomolecule Concentration)

Parameters		ACE2		Anti_NP		Aptamer	
Biomolecules concentration	Pure MNP	15 nM	30 nM	15 nM	30 nM	15 nM	30 nM
Size (nm)	51	61.5	71.8	54.4	84.2	55.14	59.55
PDI	0.117	0.22	0.262	0.118	0.3	0.194	0.181
Zeta potential (mV)	–22.9	–14.7	–12.33	–17.4	–8.36	–15.26	–16.36

### Inductively Coupled Plasma Mass Spectrometer (ICP) Analysis

To ensure that MPS signal variations arise from binding-induced relaxation changes rather than differences in magnetic loading, the iron content of both plain and conjugated MNP samples was quantified by ICP-MS. The results, summarized in Table 2, include the mean and standard deviation values of iron concentrations for each sample, calculated from two independent measurements. Because MPS signal depends on magnetic loading, the ICP-MS results in Table 2 were used to normalize all samples to the same iron mass before comparative MPS analysis. Plain MNPs exhibited an iron concentration of 3.176 ± 0.034 mg/mL, set as the reference. Upon conjugation, the Fe content of the MNPs significantly decreased due to the conjugation process and washing steps: MNPs-ACE2 retained 44.3%, MNPs-anti_NP retained 42.0%, and MNPs-Aptamer retained only 31.7% of the original Fe content. Based on these ICP-MS measurements, all samples were normalized to an identical iron mass of 5 μg Fe in a total volume of 100 μL prior to MPS measurements. The corresponding sample volumes required to achieve this normalization were calculated (Supporting Information S2): 1.6 μL for plain MNPs, 3.6 μL for MNP-ACE2, 3.75 μL for MNP-anti_NP, and 5 μL for MNP-aptamer, with the remaining volume adjusted using deionized water. This normalization ensures that any variation in the MPS signal arises from differences in hydrodynamic behavior caused by surface conjugation rather than from differences in magnetic content.

**Table 2:** Iron Concentration (mg/mL) of Protein-Conjugated MNP Samples Measured by ICP-MS^a^

Sample	Rep 1_Fe (mg/mL)	Rep 2_Fe (mg/mL)	Mean ± SD (mg/mL)	% Fe Retained
Plain MNPs	3.200	3.152	3.176 ± 0.034	100%
MNPs-ACE2	1.482	1.330	1.406 ± 0.107	44.3%
MNPs-anti_NP	1.196	1.471	1.334 ± 0.195	42.0%
MNPs-Aptamer	1.010	1.006	1.008 ± 0.003	31.7%

a Results show mean ± SD of two replicates and percentage of Fe retained relative to original MNPs.

### MPS Results

The primary objective of this study is to demonstrate the capability of the MPS instrument to detect disease biomarkers through harmonic signal modulation induced by biomolecule binding on MNPs. Previous studies have shown that surface functionalization increases the hydrodynamic size of MNPs and alters their Brownian relaxation behavior, leading to characteristic attenuation of higher-order harmonics in the MPS signal.^24,47,48^ Building upon these findings, we systematically investigated the sensitivity of the developed MPS system to protein- and aptamer-conjugated MNPs, with the goal of establishing the detection limit and evaluating biomarker-specific interactions. In MPS measurements, surface binding is expected to hinder Brownian relaxation and alter the harmonic response of MNPs. In the present measurements, the third harmonic showed stronger attenuation than the fifth harmonic. This trend is consistent with binding-induced changes in Brownian relaxation and possible reshaping of the harmonic spectrum, although the detailed mechanism governing the different responses of individual harmonics was not fully resolved in this study.^49^ Consequently, the harmonic ratio R_53_ (fifth/third) increases monotonically with surface loading and serves as a robust, mass-normalized indicator of binding-induced relaxation delay.^20,24,28,29^

Figure 3 summarizes the MPS responses of MNPs conjugated with varying concentrations of biomolecules including ACE2, anti_NP, and Aptamer. For all three systems, increasing biomolecule concentration produced a concentration-dependent attenuation of both the third and fifth harmonic amplitudes, with the strongest suppression consistently observed in the third harmonic under the present measurement conditions. Statistical analysis (one-way ANOVA followed by pairwise *t* tests) confirmed significant differences between concentration groups (*p < 0.05 to ***p < 0.001). For ACE2- and anti_NP-conjugated MNPs, detectable attenuation was observed at concentrations as low as 1 nM, while aptamer-conjugated MNPs exhibited a detection threshold of 12 nM. The higher detection limit observed for aptamer-conjugated MNPs compared to protein-conjugated systems arises from the smaller hydrodynamic footprint and reduced rotational drag imposed by aptamers, requiring higher surface coverage to induce comparable Brownian relaxation delays.

In Figure 3C,F,I, the harmonic ratio R_53_ (fifth/third) increased with increasing surface loading, is consistent with Brownian relaxation delay arising from enlarged hydrodynamic size and restricted rotational mobility. This behavior is in agreement with theoretical predictions and earlier experimental reports on MPS-based biosensing.^20,24,28^ These results confirm that MPS provides nanomolar-level sensitivity for diverse classes of biomolecules and is well suited for early stage biomarker detection.

To assess the ability of the MPS to detect biologically relevant molecular recognition events for realistic diagnostic conditions, targeted binding experiments were performed in which a fixed amount of primary ligand (ACE2, anti_NP, or AS1411 aptamer) was conjugated on the MNP surface, followed by exposure to increasing concentrations of the corresponding target proteins (RBD, H1N1 NP, and nucleolin, respectively). In contrast to primary conjugation, secondary binding forms multiprotein complexes, producing a substantially larger hydrodynamic volume and enhanced Brownian relaxation delay. Under the present measurement conditions, this effect was observed as stronger attenuation of the third harmonic amplitude and a corresponding increase in the harmonic ratio R_53_, which serves as a hydrodynamic relaxation metric with reduced sensitivity to total nanoparticle mass compared with absolute harmonic amplitudes.

For ACE2-conjugated MNPs, stepwise addition of SARS-CoV-2 RBD resulted in a robust and monotonic attenuation of the third harmonic and a corresponding increase in R_53_ (Figure 4A–C). Similar concentration-dependent behavior was observed for anti_NP-conjugated MNPs exposed to H1N1 nucleoprotein (Figure 4D–F), demonstrating that the MPS platform can sensitively resolve antibody–antigen interactions relevant to influenza diagnostics. Aptamer-conjugated MNPs interacting with nucleolin likewise exhibited clear harmonic attenuation and an increasing R_53_ trend (Figure 4G–I), confirming effective nucleic-acid-mediated target recognition. Across all systems, statistically significant signal changes were detected at target concentrations as low as 5 nM, defining the effective detection limit for secondary binding under the present measurement conditions.^50^ A deeper biophysical insight reveals that the hydrodynamic modulation of MNPs is not only a function of molecular binding but also influenced by protein size and surface density. The smaller size of the RBD protein (26.5 kDa) compared to H1N1 (56.6 kDa) enables a higher number of binding events per nanoparticle at equivalent molar concentrations, thereby inducing greater suppression of Brownian rotation and enhanced harmonic attenuation.^28^

**Figure 4:**
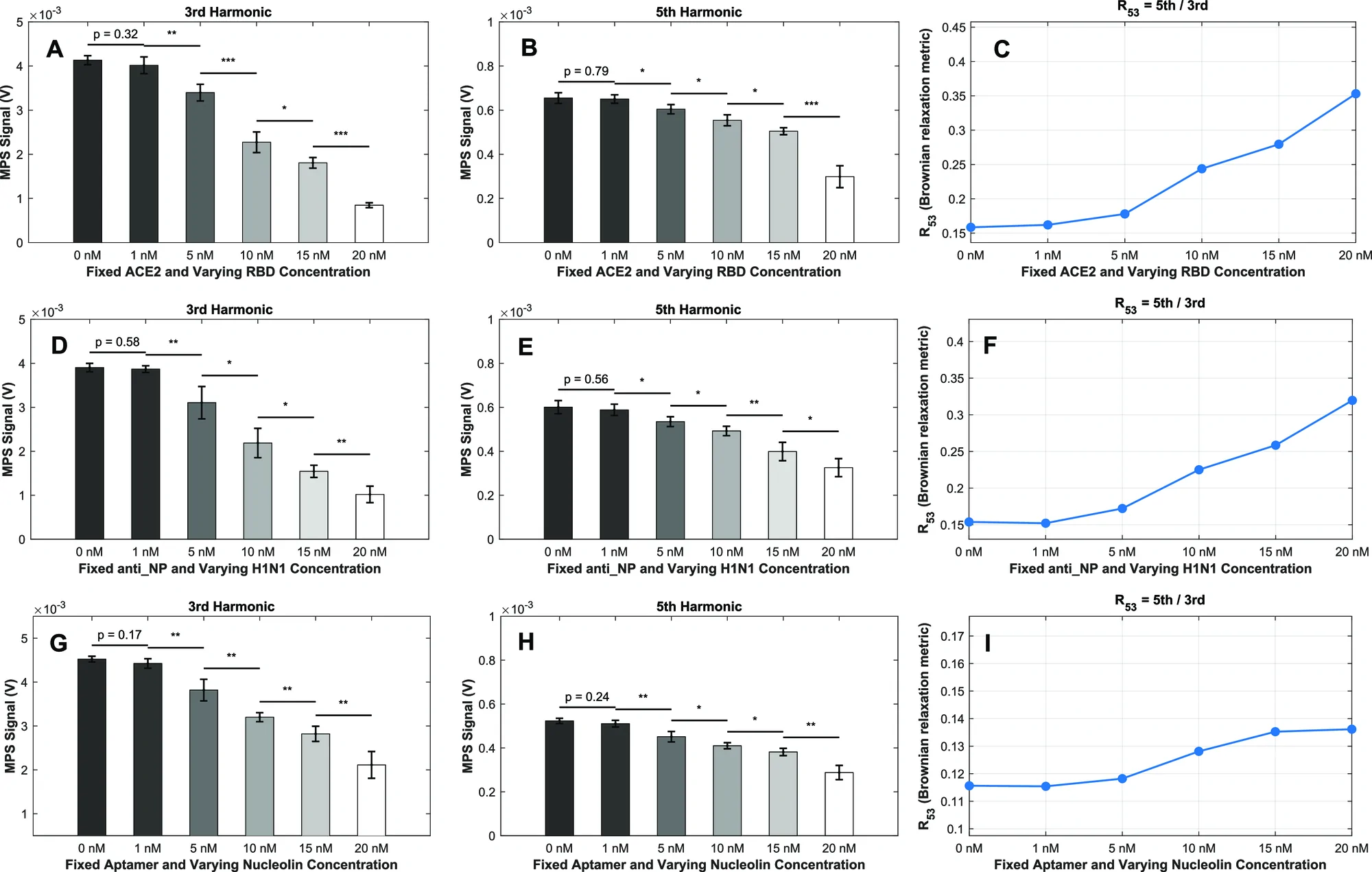
MPS detection of target binding protein–protein and aptamer-protein interactions. For each system (ACE2-RBD, anti_NP-H1N1, AS1411-nucleolin), the third and fifth harmonic amplitudes decrease systematically with increasing target concentration, indicating additional hydrodynamic drag following specific ligand–target binding. The harmonic ratio R_53_ *=* fifth/third rises monotonically, providing partially normalized Brownian relaxation metric that captures the progression of secondary complex formation with reduced dependence on nanoparticle mass. These results demonstrate the capability of MPS to resolve target binding events down to the low-nanomolar range. Error bars indicate standard deviations across replicate measurements with statistical significance indicated (*p < 0.05, **p < 0.01, ***p < 0.001).

Control experiments were performed with unconjugated COOH-functionalized SHP-25 MNPs incubated directly with RBD, H1N1 nucleoprotein, or nucleolin showed no comparable target-specific MPS response, supporting that the signal changes observed in the functionalized systems primarily arise from specific ligand-target recognition rather than target addition alone. Experimental details are provided in Supporting Information Scheme S4 and results are shown in Figure S2. To further validate specificity, the relative Brownian-relaxation metric R_53_ was compared between the targeted binding condition and the nonspecific control condition for all three biomarker systems. As shown in Figure 5, the specific ligand–target systems exhibited clear concentration-dependent increases in R_53_, whereas direct incubation of the same targets with unconjugated COOH-functionalized SHP-25 MNPs resulted in only minimal variation. These results support that the main MPS response arises from specific biomolecular recognition rather than nonspecific target addition.

**Figure 5:**
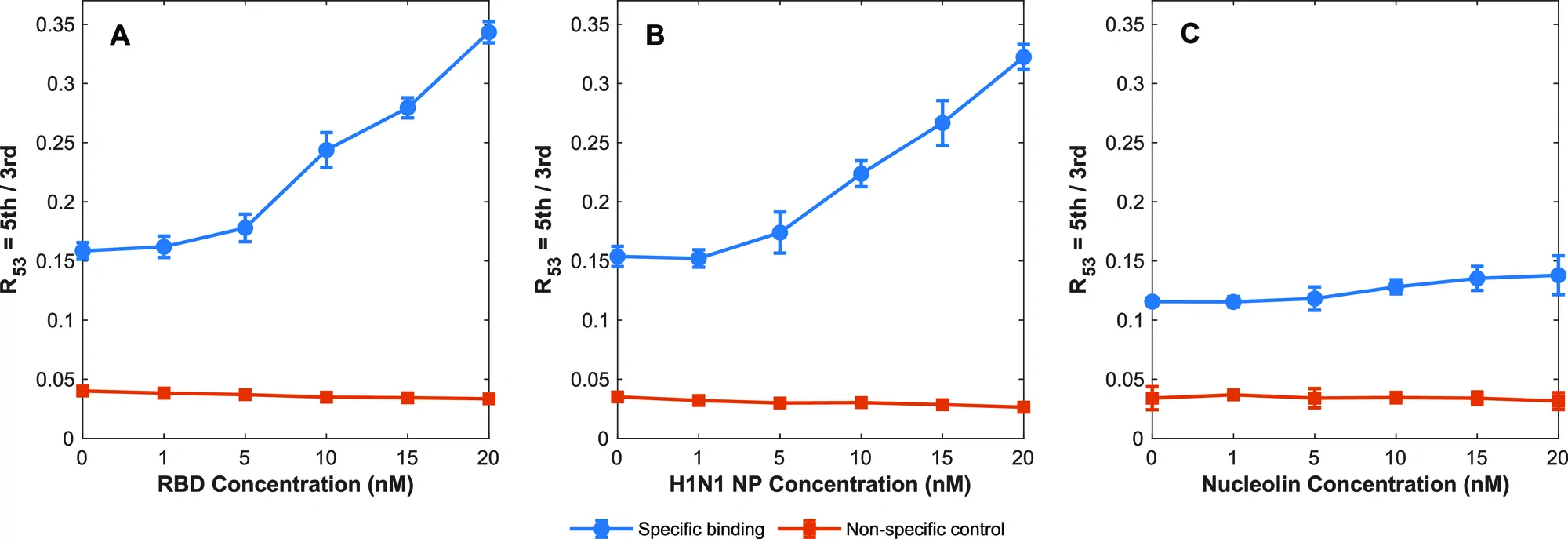
Comparison of the relative Brownian-relaxation metric R_53_ (fifth/third) for (A) RBD, (B) H1N1 nucleoprotein, and (C) nucleolin under targeted and nonspecific control conditions. Targeted systems showed clear concentration-dependent increases in R_53_, whereas direct incubation of the same targets with unconjugated COOH-functionalized SHP-25 MNPs produced only minimal variation. These results support that the measured response is primarily governed by specific biomolecular recognition.

### Comparison of Monoclonal and Polyclonal Antibodies in MPS Measurements

The choice of biorecognition element, particularly monoclonal versus polyclonal antibodies, significantly influences MPS signal behavior and assay performance. Comparative analysis revealed that monoclonal and polyclonal antibody conjugated to MNPs produce distinct harmonic responses due to differences in binding homogeneity, epitope selectivity, and aggregation propensity.

Monoclonal antibodies (SARS-CoV-2 RBD, and influenza A H1N1 nucleoprotein, NP I116M), owing to their single-epitope specificity, promote uniform, one-to-one binding events on the conjugated-MNP surface, leading to a controlled increase in hydrodynamic volume and concentration-dependent attenuation of higher-order harmonics in MPS measurements (Figure 6). In contrast, polyclonal antibodies (polyclonal spike RBD antibody, rabbit IgG, and polyclonal influenza A nucleoprotein antibody, rabbit IgG, antigen-affinity purified) bind multiple epitopes on the same antigen, which can lead to heterogeneous binding configurations and may promote interparticle cross-linking and aggregation-like behavior. For both RBD (SARS-CoV-2) and H1N1 (Influenza) detection (Figure 6C,D,F,G), this behavior produces a rapid suppression of the third harmonic, reflecting strong inhibition of Brownian rotation and suggestive of clustering-related effects. Consequently, the harmonic ratio R_53_ (fifth/third) diverges more rapidly for polyclonal systems than for monoclonal systems (Figure 6E,H), suggesting a transition from predominantly Brownian-dominated responses toward aggregation-like or clustering-associated signal changes.

**Figure 6:**
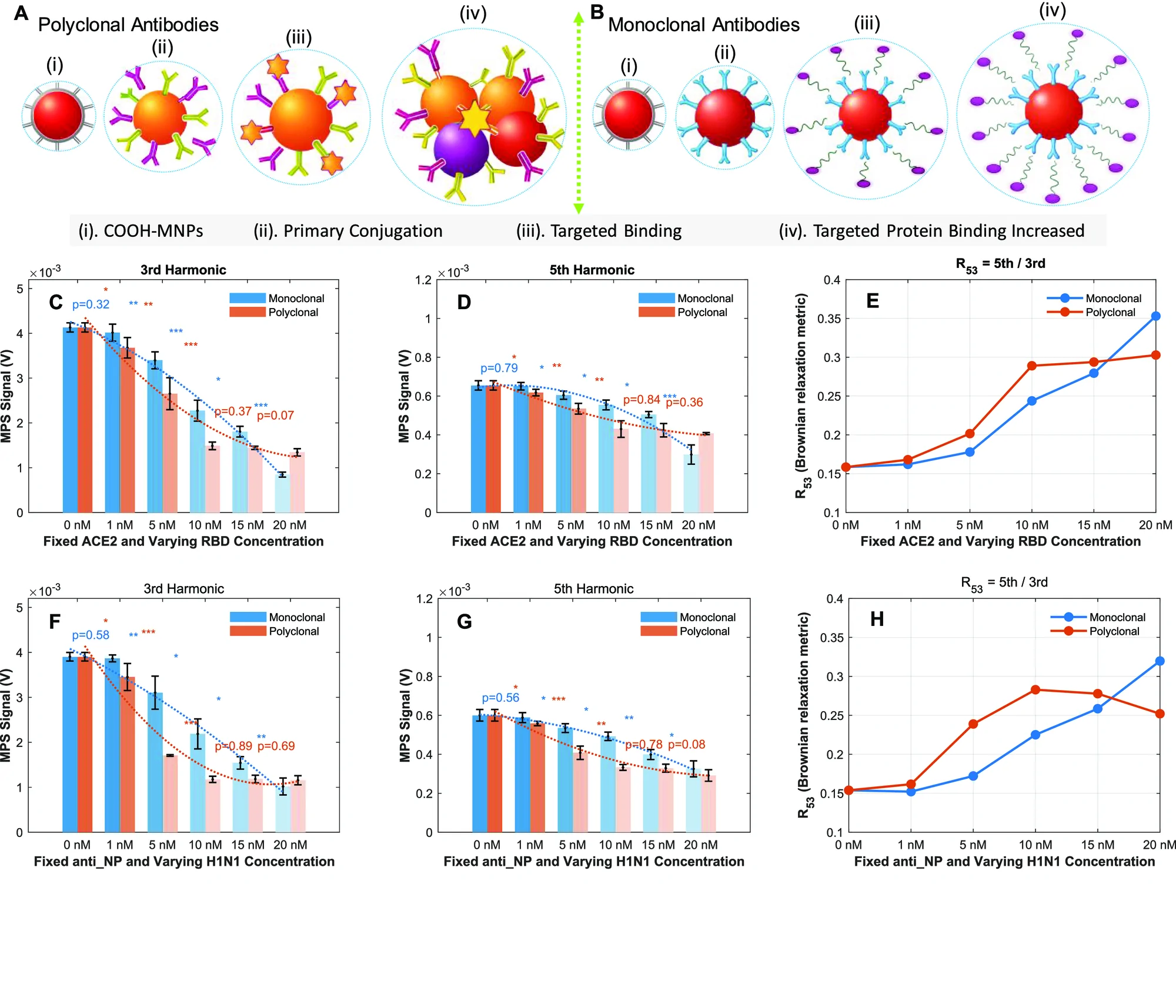
Comparative MPS response of monoclonal- and polyclonal antibody–conjugated MNPs for SARS-CoV-2 RBD and influenza A (H1N1) detection. (A) Schematic of polyclonal antibody-functionalized MNPs, (B) Schematic of monoclonal antibody-functionalized MNPs. (C, D) show the concentration-dependent attenuation of the third and fifth harmonic amplitudes for monoclonal and polyclonal RBD systems, respectively, highlighting the gradual harmonic decay associated with uniform binding in monoclonal assays and the rapid suppression of the third harmonic observed in polyclonal assays, which is consistent with aggregation-like or clustering-associated effects. Panels (E) and (H) present the corresponding harmonic ratio R_53_ (fifth/third), illustrating the faster divergence of polyclonal systems suggestive of clustering-driven Brownian relaxation inhibition. Panels (F) and (G) summarize the harmonic responses for H1N1, demonstrating consistent trends across viral targets. Error bars indicate standard deviations across replicate measurements with statistical significance indicated (*p < 0.05, **p < 0.01, ***p < 0.001).

The higher specificity of monoclonal antibodies reduces nonspecific binding and cross-reactivity, which is critical for reliably distinguishing closely related viral targets such as coronaviruses and influenza strains, thereby enhancing assay reproducibility, quantitative accuracy, and dynamic range in MPS-based biosensing. Whereas polyclonal antibodies may be advantageous for rapid screening applications and better sensitivity where signal amplification through multivalent interactions is desired. The apparent maximum in the polyclonal response around 10 nM is interpreted as possible early saturation consistent with aggregation-like behavior, where multivalent cross-linking may restrict Brownian rotation rapidly and compresses the dynamic response range.^51^

### Quantitative Analysis of Binding-Induced MPS Signal Attenuation

To enable quantitative comparison across biorecognition elements and targets, third-harmonic amplitudes were normalized to their respective controls following the protocol described in Supporting Information S3, and the mean attenuation values are summarized in Figure 7. During primary conjugation (Figure 7A–C), attenuation increased monotonically with biomolecule concentration and approached near to saturation at higher loadings, consistent with progressively hindered Brownian rotation as surface coverage increased. Among the tested biorecognition elements, ACE2-conjugated MNPs reached the highest attenuation (∼69% at 16 nM), followed by anti_NP (∼61%) and AS1411 aptamer (∼55%) at their maximum concentrations, while the steep initial rise indicates that the MPS signal is highly responsive to early stages of surface loading. For secondary target binding (Figure 7D–F), attenuation increased more steeply than for primary conjugation alone, reflecting formation of larger surface complexes upon target attachment. In particular, ACE2-RBD (monoclonal) binding exceeded ∼80% attenuation at 25 nM RBD, whereas anti_NP-H1N1­(monoclonal) and AS1411-nucleolin reached ∼77% and ∼59%, respectively. In contrast, polyclonal secondary binding to the same targets (Figure 7G–I) resulted in a more rapid initial attenuation and earlier saturation compared with monoclonal systems.

**Figure 7:**
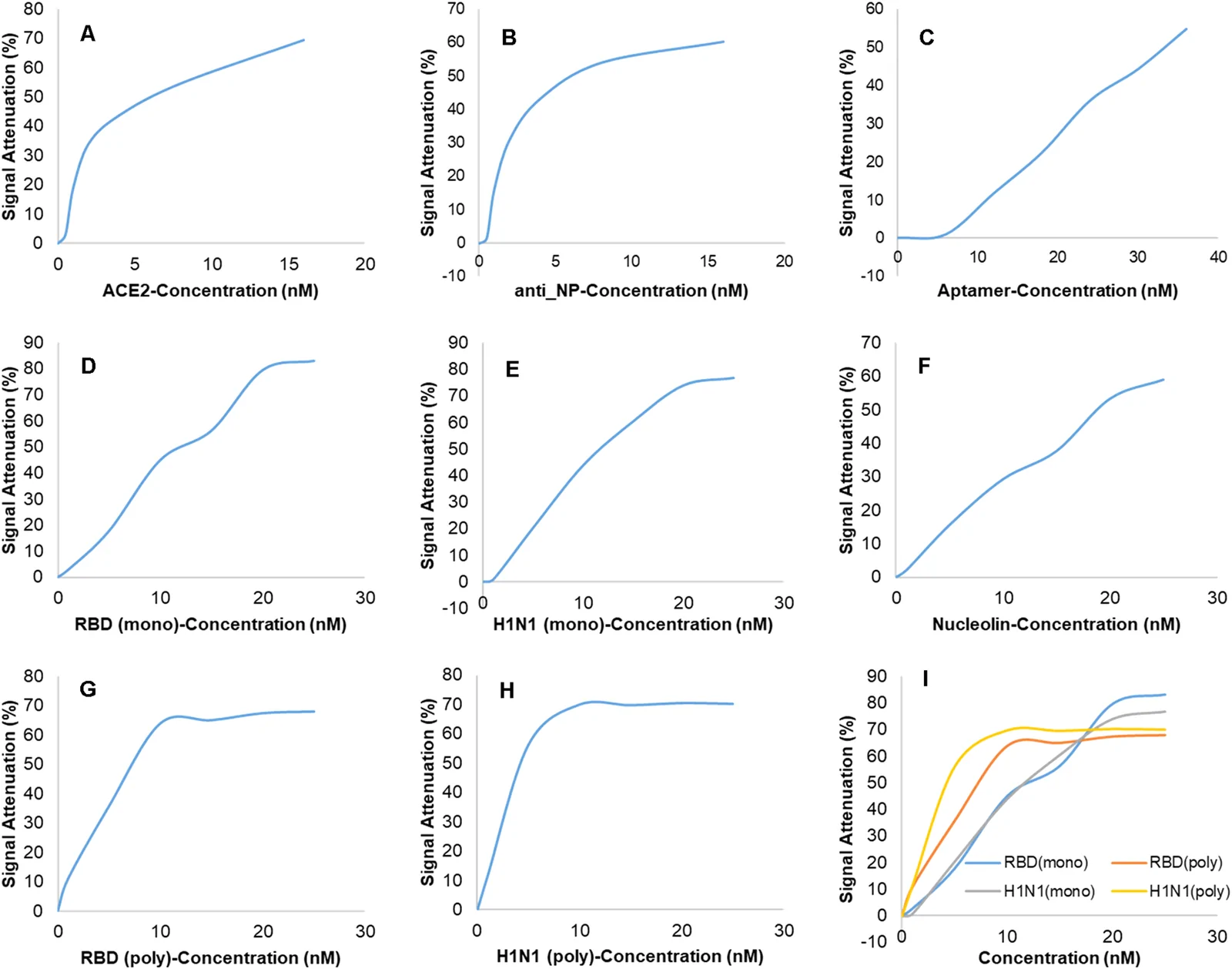
Quantitative analysis of MPS signal attenuation induced by biomolecular binding. Signal attenuation (%) calculated from normalized harmonic amplitudes for primary binding of (A) ACE2, (B) anti_NP, and (C) AS1411 aptamer to MNPs, and for secondary (targeted) binding of (D) RBD (monoclonal), (E) H1N1 nucleoprotein (monoclonal), (F) nucleolin, (G) RBD (polyclonal), and (H) H1N1 nucleoprotein (polyclonal) to the corresponding conjugated MNPs. (I) shows the comparison plot for monoclonal and polyclonal binding. Attenuation increases monotonically with biomolecule concentration, reflecting progressive suppression of Brownian relaxation upon surface binding for the case of monoclonal antibodies, whereas in polyclonal systems, early saturation is consistent with aggregation-like or clustering-associated effects that may limit further concentration-dependent signal changes.

For polyclonal RBD and H1N1 binding, maximum attenuation values of ∼68% and ∼70%, respectively, were observed, beyond which further increases in target concentration produced minimal additional signal change. This behavior suggests that polyclonal binding may promote faster saturation consistent with aggregation-like or clustering-associated effects, while yielding a lower overall attenuation than monoclonal binding. Measurable attenuation was observed from the lowest tested target concentrations (5 nM in this study), defining an effective detection threshold for secondary binding under the present measurement conditions and highlighting that biomarker recognition produces a stronger relaxation signature than nonspecific surface loading.^30^ Because direct postbinding aggregation measurements were not performed in this study, the clustering or aggregation-related interpretation should be considered suggestive rather than definitive and will require validation by postbinding DLS, TEM, or microscopy in future work.

This work demonstrates a high-sensitivity MPS platform for label-free biosensing based on Brownian relaxation, in which biomolecular binding increases hydrodynamic drag and suppresses nanoparticle rotational dynamics. The resulting stepwise signal response enables MPS readouts to be interpreted both as a verification tool for nanoparticle probe formation and as a quantitative modality for specific biomolecular recognition. Assay performance is shown to depend strongly on the choice of biorecognition element. Monoclonal antibody-induced binding exhibited gradual, reproducible harmonic attenuation and a well-defined dynamic range, consistent with relatively uniform and predominantly one-to-one binding, whereas polyclonal antibody systems displayed signal distortion suggestive of aggregation-like or clustering-associated effects. These differences arise from intrinsic limits in nanoparticle surface functionalization: only a finite number of binding sites are available on each MNP, beyond which additional biomolecules remain unbound and do not further affect Brownian relaxation.^41^ In monoclonal systems, surface coverage increases in a controlled manner until saturation is reached, while in polyclonal systems multivalent binding can bridge neighboring MNPs, leading to early aggregation and premature signal saturation.

To better position the present study within the broader biosensing literature, representative comparisons with previously reported MPS-based assays and established diagnostic methods are provided in Supporting Information Tables S4 and S5. These comparisons indicate that the present platform should be regarded as a complementary biosensing modality for ELISA, PCR, or flow cytometry.^52,53^ Its main strengths lie in rapid magnetic readout, reduced workflow complexity under the tested assay conditions, nanogram-level iron-mass sensitivity, and applicability across multiple recognition modes. Relative to many prior MPS biosensing studies, the present work further emphasizes instrument-level sensitivity enhancement through signal integration, and harmonic-ratio analysis, together with a two-stage detection strategy involving probe conjugation followed by target recognition.

In this study, monoclonal antibodies were used for primary conjugation, while monoclonal or polyclonal ligands were introduced only during secondary binding. This strategy minimizes aggregation during probe preparation and enables controlled evaluation of target-specific interactions. As a result, combining a highly sensitive MPS platform with monoclonal antibody-based recognition yields enhanced specificity, quantitative reliability, and operational flexibility for biomarker detection. Compared with many previously reported MPS biosensing strategies that rely on aggregation-driven signal amplification,^20,24,28,29,43,54^ the present approach emphasizes controlled molecular recognition coupled with instrument-level sensitivity. This design enables reliable detection of early stage biomolecular interactions and maintains consistent performance across multiple biologically relevant systems, including protein–protein and aptamer-protein binding.

A limitation of the present study is that sensing performance was evaluated under simplified buffer conditions rather than in complex biological matrices such as serum, plasma, or saliva, where protein adsorption, biomolecular fouling, and protein corona formation may alter nanoparticle behavior and influence magnetic relaxation responses.^55^ Such effects can occur even for surface-functionalized nanoparticles, although surface functionalization may modulate their extent.^56^ In this context, monoclonal antibody-functionalized MNPs were used to improve target specificity and reduce nonspecific interactions under the present assay conditions. The harmonic ratio R_53_ may also provide partial analytical robustness against signal variations unrelated to specific binding, although this remains to be verified experimentally in realistic biological media.^57^

Iron-mass normalization by ICP-MS was used in this study as part of analytical validation to ensure that differences in MPS responses originated from binding-induced changes in nanoparticle relaxation rather than variation in nanoparticle concentration. This control enabled rigorous comparison across plain and conjugated formulations and supported sensitivity analysis under defined conditions.^33^ However, this does not mean that routine iron quantification would be required in practical biosensing applications. In a translational assay format, the concentration of conjugated MNPs would be predefined and standardized as part of the sensing reagent, while the unknown parameter would be the target biomarker concentration. In such cases, calibration could rely on fixed-dose reagent formulations and pre-established response curves, consistent with established nanoparticle assay principles.^58^ In addition, the harmonic ratio R_53_ may provide partial intrinsic normalization against moderate variation in nanoparticle amount, while internal standards could be used when needed. Future studies will therefore evaluate matrix effects, confirm specificity in clinically relevant fluids, and further assess translational applicability. Critical-offset approaches such as COMPASS^30^ may offer additional robustness for unknown or variable-concentration samples and represent a valuable future extension of the present platform.

## Conclusion

In this work, we developed and demonstrated a highly sensitive MPS-based biosensing strategy that integrates instrument-level sensitivity with a detection strategy consisting of nanoparticle probe conjugation followed by specific target binding. The system achieved magnetic sensitivities of 1 ng Fe for Synomag and 3 ng Fe for SHP-25 nanoparticles. Primary conjugation supported detection limits of 1 nM (100 fmol) for ACE2 and anti_NP and 12 nM (1.2 pmol) for the AS1411 aptamer, while secondary target recognition consistently produced statistically significant signal attenuation at 5 nM (500 fmol) across investigated monoclonal systems including ACE2-RBD, anti_NP-H1N1 nucleoprotein, and AS1411-nucleolin interactions. Quantitative harmonic analysis showed that normalized attenuation of the third harmonic increased monotonically during primary conjugation, reflecting progressive suppression of Brownian relaxation as hydrodynamic size increased, whereas secondary target recognition produced stronger relaxation effects due to the formation of larger biomolecular complexes. The harmonic ratio R_53_ (fifth/third) further enhanced detection robustness by providing a partially normalized metric with reduced dependence on nanoparticle mass, improving discrimination between primary conjugation and specific biomolecular recognition. Importantly, the presented strategy enhances MPS biosensing sensitivity and specificity without increasing assay complexity or requiring washing or labeling steps, establishing its suitability for quantitative biomarker detection. Overall, this work positions MPS as a versatile and scalable platform with potential applications in early-stage diagnostics, viral detection, and cancer biomarker screening. While the present study establishes performance under controlled conditions, future efforts will focus on selectivity against nontarget interferents and validation in complex biological matrices to further advance translational applicability.

## Supplementary Material



## Notes

Additional experimental details; control experiments; frequency-response data; qualitative evaluation of protein conjugation to MNPs using the Bradford colorimetric assay; control MPS measurements of unconjugated COOH-functionalized SHP-25 MNPs; dialysis process; calculation protocol for signal attenuation samples; details for Fourier-transform infrared spectroscopy (FT-IR) and dynamic light scattering (DLS) analysis using SHP-25 MNPs, and detection of secondary binding (antibody/target interaction) (PDF)
