# Supplementary material for: High-Sensitivity Magnetic Particle Spectroscopy Platform for Precise, Specific, and Rapid Detection of Infectious Disease and Cancer-Related Biomarkers
Source: ACS Meas Sci Au. 2026 Jun 29;6(4):1023–37. doi: 10.1021/acsmeasuresciau.6c00032 (PMC13495727; doi:10.1021/acsmeasuresciau.6c00032)
Supplement: Supplementary file 1 [file tg6c00032_si_001.pdf]

Supplementary Information

High-Sensitivity Magnetic Particle Spectroscopy Platform for Precise, Specific and Rapid Detection of Infectious Disease and Cancer-Related Biomarkers

Hafiz Ashfaq Ahmad[1], Ali Dinari[*1,2], Minh Phu Bui[1], Seungjun Oh[1], Masoud Rezaie[3], Shahla Kianamiri[4], and Jungwon Yoon[*,1]

[1] *Department of AI Convergence, Gwangju Institute of Science and Technology, Gwangju, 61005, Republic of Korea*

[2]*Department of Medical Physics, M. Smoluchowski Institute of Physics, Jagiellonian University, ul. S. Łojasiewicza 11, 30-348, Krakow, Poland.*

[3]*Department of Chemistry, Jagiellonian University, Gronostajowa 2, 30-387 Cracow, Poland*

[4]*Department of Nanotechnology, Agricultural Biotechnology Research Institute of Iran (ABRII), Agricultural Research, Education and Extension Organization (AREEO), 31359-33151 Karaj, Iran*

[*]*Email:* jyoon@gist.ac.kr

[*1,2]*Email:* ali.dinari606@gmail.com

Scheme S1: Dialysis Process:

The Tris–glycine buffer was replaced with MES buffer (20 mM MES, 200 mM NaCl, pH 6.5). This step was critical for ensuring high coupling efficiency and minimizing nonspecific interactions. To this end, the protein powders, as supplied by the manufacturer, were first dissolved in 400 µL of deionized water to obtain a concentration of 0.25 mg/mL. From each protein stock solution, 750 µL of a 0.1 mg/mL sample was prepared in deionized water. Subsequently, 450 µL of 20 mM MES buffer (containing 200 mM NaCl, pH 6.5) was added to each solution, and the mixtures were transferred into dialysis bags. Dialysis was carried out by exchanging the external water every 1 hour for three cycles, followed by water replacement every 3 hours for an additional four cycles. To verify the presence of proteins after dialysis and to confirm their retention following conjugation with MNPs, a Bradford protein assay was performed. This assay was not intended to determine the efficiency of the EDC/NHS coupling reaction but rather to confirm the presence of protein before and after the conjugation process. For the assay, six test tubes were prepared. Tube 1 contained unmodified MNPs diluted in water (100 µL), and tube 2 contained Bradford reagent alone (100 µL); both served as negative controls, displaying only their intrinsic colors. Tube 3 contained a mixture of unmodified MNPs and Bradford reagent, which showed no significant color change, confirming the absence of free or nonspecifically adsorbed proteins on the nanoparticles. Tube 4, consisting of free protein mixed with Bradford reagent, produced a distinct blue color, serving as a positive control for protein–dye binding. Tubes 5 and 6 contained MNPs conjugated with H1N1 protein (Tube 5) and RBD protein (Tube 6), respectively. Both samples exhibited clear shifts toward blue coloration, confirming the presence and retention of proteins on the nanoparticle surface following purification (Figure S1). The Bradford assay was not applied to aptamer-conjugated MNPs, as this method relies on interactions with aromatic and basic amino acid residues and does not reliably detect nucleic acids such as DNA aptamers.

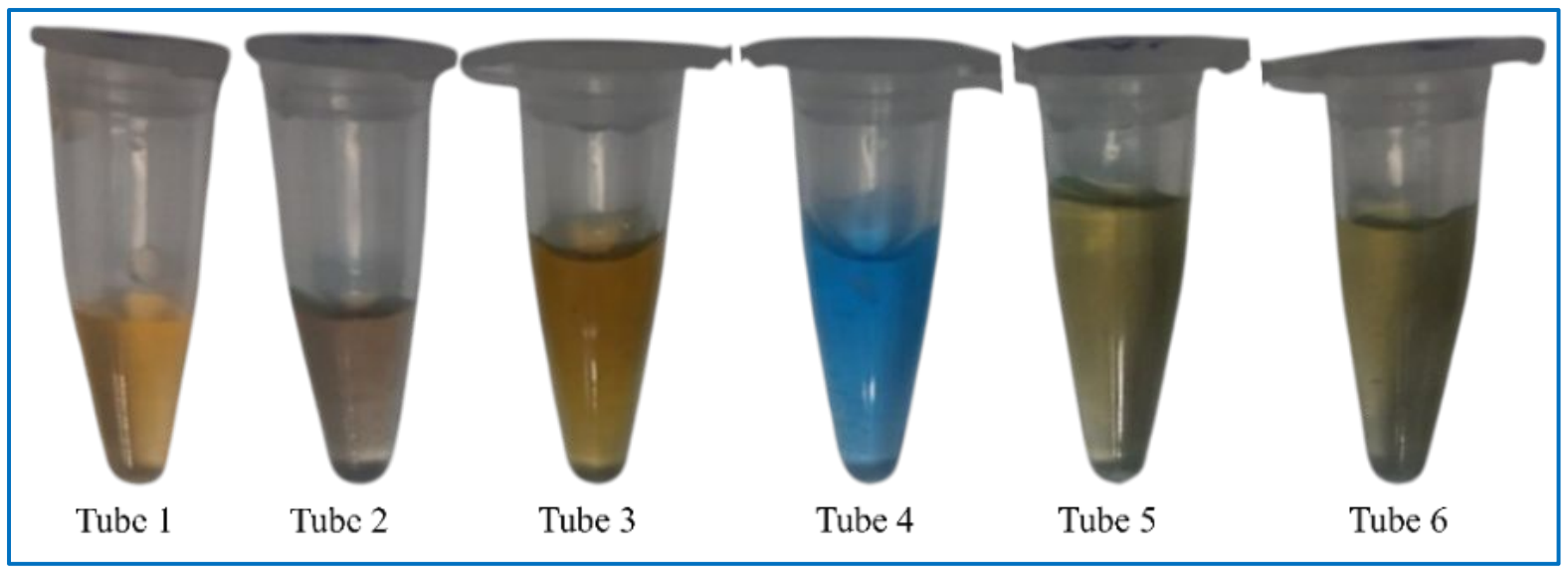

Figure S1. Qualitative evaluation of protein conjugation to MNPs using the Bradford colorimetric assay. Tube 1: unmodified MNPs in water (negative control); Tube 2: Bradford reagent alone (negative control); Tube 3: unmodified MNPs mixed with Bradford reagent, showing no color change and confirming the absence of protein; Tube 4: free protein mixed with Bradford reagent, showing a strong blue color as a positive control; Tubes 5 and 6: MNPs conjugated with H1N1 and RBD respectively, both displaying a blue shift indicative of the presence of protein.

Table S1: Samples details for Fourier-transform infrared spectroscopy (FT-IR) and dynamic light scattering (DLS) analysis using SHP-25 MNPs. In all samples, the MNPs concentration is 5 μg in 100 μL.

Sample Type	Sample description	Biomolecule concentration	No. of samples	Target type
MNP-ACE2-1	MNPs conjugated with ACE2	15 nM	06	Viral (COVID-19)
MNP-ACE2-2	MNPs conjugated with ACE2	30 nM	06	
MNP-anti_NP-1	MNPs conjugated with anti_NP	15 nM	06	Viral (Influenza)
MNP-anti_NP-2	MNPs conjugated with anti_NP	30 nM	06	
MNP-Aptamer-1	MNPs conjugated with Aptamer	15 nM	06	Cancer (Breast)
MNP-Aptamer-2	MNPs conjugated with Aptamer	30 nM	06	
MNPs	Plain MNPs (no conjugation)	–	06	Reference/Control

Table S2: Here are concise sample details to test the Sensitivity for the conjugated protein with MNPs. All samples were Fe-normalized before MPS.

Assay target (antigen/biomarker)	Description	Total volume (µL)	Protein concentration (nM)	Amount of protein (fmole)	Fe mass (µg)	No. of samples
ACE2	Plain MNPs (control)	100	0	0	5	10
	MNPs + ACE2	100	0.5	50	5	10
	MNPs + ACE2	100	1	100	5	10
	MNPs + ACE2	100	2	200	5	10
	MNPs + ACE2	100	4	400	5	10
	MNPs + ACE2	100	8	800	5	10
	MNPs + ACE2	100	16	1600	5	10
Anti_NP antibody	Plain MNPs (control)	100	0	0	5	10
	MNPs + anti_NP	100	0.5	50	5	10
	MNPs + anti_NP	100	1	100	5	10
	MNPs + anti_NP	100	2	200	5	10
	MNPs + anti_NP	100	4	400	5	10
	MNPs + anti_NP	100	8	800	5	10
	MNPs + anti_NP	100	16	1600	5	10
AS1411 aptamer	Plain MNPs (control)	100	0	0	5	10
	MNPs + Aptamer	100	6	600	5	10
	MNPs + Aptamer	100	12	1200	5	10
	MNPs + Aptamer	100	18	1800	5	10
	MNPs + Aptamer	100	24	2400	5	10
	MNPs + Aptamer	100	30	3000	5	10
	MNPs + Aptamer	100	36	3600	5	10

Table S3: Detection of secondary binding (antibody/target interaction): Primary conjugation is fixed for all Step-2 baselines; Fe mass is normalized before MPS. Readout focuses on 3rd-harmonic attenuation and 5th- harmonic recorded.

Secondary / targeted biomarker)	Description	Concentration of surface biomolecule (nM)	Target protein concentration (nM)	Sample volume (µg)	Amount of target protein (fmole)	Fe mass (µg)	No. of samples
RBD (SARS-CoV-2 spike protein)	Pre-conjugated MNPs baseline: MNPs-RBD	8	0	100	0	5	10
	MNPs + ACE2 + RBD	8	1	100	100	5	10
	MNPs + ACE2 + RBD	8	5	100	500	5	10
	MNPs + ACE2 + RBD	8	10	100	1000	5	10
	MNPs + ACE2 + RBD	8	15	100	1500	5	10
	MNPs + ACE2 + RBD	8	20	100	2000	5	10
H1N1 (Influenza A nucleoprotein)	Pre-conjugated MNPs baseline: MNPs-H1N1	8	0	100	0	5	10
	MNPs + anti_NP + H1N1	8	1	100	100	5	10
	MNPs + anti_NP + H1N1	8	5	100	500	5	10
	MNPs + anti_NP + H1N1	8	10	100	1000	5	10
	MNPs + anti_NP + H1N1	8	15	100	1500	5	10
	MNPs + anti_NP + H1N1	8	20	100	2000	5	10
Nucleolin	Pre-conjugated MNPs baseline: MNPs-Aptamer	24	0	100	0	5	10
	MNPs + Aptamer + Nucleolin	8	1	100	100	5	10
	MNPs + Aptamer + Nucleolin	8	5	100	500	5	10
	MNPs + Aptamer + Nucleolin	8	10	100	1000	5	10
	MNPs + Aptamer + Nucleolin	8	15	100	1500	5	10
	MNPs + Aptamer + Nucleolin	8	20	100	2000	5	10

Scheme S2: To prepare 100 µL of sample containing exactly 5 µg of Fe, using:

Volume Calculation Formula:

$$Volume = \frac{Required\ Concentration\ (\mu g)}{ICP\ Determind\ Concentration\ (\mu g/\mu L)}$$

$$Water\ Volume = Total\ Sample\ Required\ Volume - Volume\ of\ MNPs\ or\ conjugates$$

Scheme S3: Calculation Protocol for Signal Attenuation

1. Data extraction: Raw harmonic amplitudes for each experimental condition were obtained directly from the MPS output (3rd and 5th harmonics). Each data point represents the average of three independent measurements.

2. Normalization: All signals were normalized to appropriate reference controls to eliminate variability due to instrument gain or iron concentration. For each experimental condition, the normalized relative signal was calculated as:

$$\text{Relative Signal} = \frac{S_{sample}}{S_{control}}$$

   Here:

   - For primary binding, $S_{control}$ corresponds to plain (unmodified) MNPs, and S_{sample} denotes protein- or aptamer-conjugated MNPs (ACE2, anti_NP, or Aptamer).

   - For secondary binding, $S_{control}$ represents pre-conjugated MNPs, and S_{sample} refers to the post-binding state after exposure to RBD, H1N1, or nucleolin targets.

3. Percentage attenuation: The relative signal decrease was expressed as a percentage attenuation:

$$\%\ \text{Attenuation} = (1 - \frac{S_{sample}}{S_{control}}) \times 100$$

Scheme S4: Control experiments for nonspecific target addition

To assess whether target proteins alone could alter the MPS signal in the absence of specific surface functionalization, control measurements were performed using unconjugated COOH-functionalized SHP-25 MNPs. RBD, H1N1 nucleoprotein, and nucleolin were added directly to the MNP suspensions at the same concentration range used in the secondary binding experiments (0, 1, 5, 10, 15, and 20 nM), while maintaining identical iron loading (5 µg Fe in 100 µL). Measurements were conducted immediately after incubation and without washing to conservatively assess possible nonspecific adsorption effects. As shown in Figure S2, only minor variations were observed in the 3rd and 5th harmonic amplitudes and in the R_{53} ratio, with no response pattern comparable to that obtained in the specifically functionalized systems. These control results support that the main MPS signal changes reported in this study are primarily associated with specific ligand-target recognition.

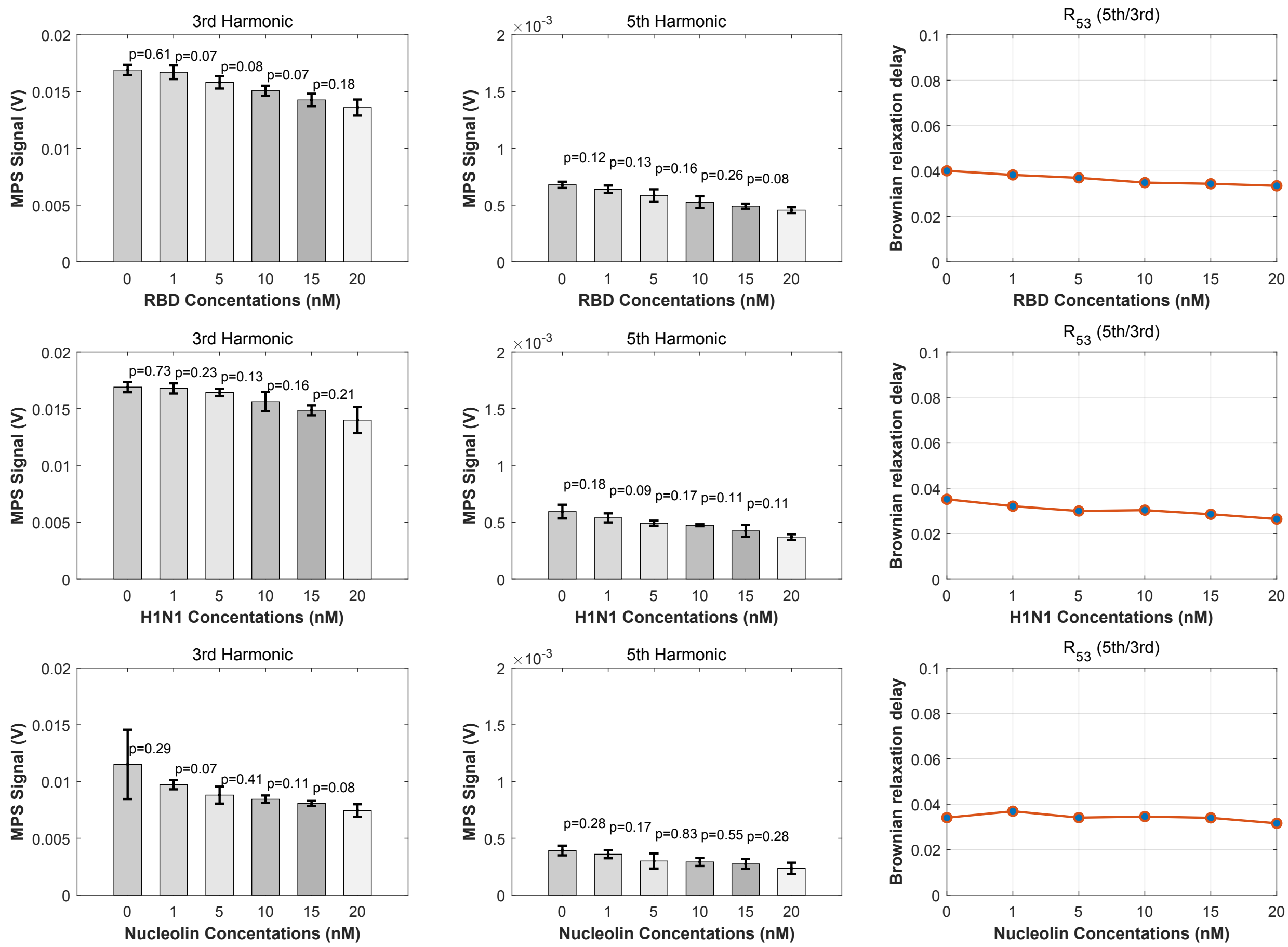

Figure S2. Control MPS measurements of unconjugated COOH-functionalized SHP-25 MNPs after direct incubation with RBD, H1N1 nucleoprotein, and nucleolin at 0, 1, 5, 10, 15, and 20 nM under identical iron loading conditions (5 µg Fe in 100 µL). The 3rd harmonic, 5th harmonic, and harmonic ratio R_{53} (5th/3rd) show only minor variations and no response pattern comparable to that observed in the specifically functionalized ligand-target systems, indicating that target addition alone does not produce the main binding-induced MPS response under the present buffer-based conditions.

Table S4. Comparison of reported MPS-based biosensing approaches with the present work

Paper	Method used	MPS-specific innovation	LOD (iron mass / MNP amount)	LOD (protein / target concentration)	Target binder type (mono/poly/other)	Key distinction relative to the present work
**Zhang et al., Biosensors and Bioelectronics, 2013** (MSB) [1]	MSB sensing using functionalized MNPs	Harmonic ratios as concentration-independent readout	ng quantities of iron within 5 s. But didn't mention any value.	Streptavidin–biotin: <150 pM (0.075 pmol); thrombin: 4 nM (2 pmol); DNA: 100 pM (0.05 pmol)	DNA aptamers (anti-thrombin)	Present work achieves a lower Fe LOD (1 ng Fe) using gradiometer-based SNR enhancement and extends MPS to disease-relevant biomarker pairs with two-stage detection
**Chugh et al., J. Phys. Chem. C, 2021 [2]**	One-stage lock-in MPS with bead-based bioassay	Passive excitation feedthrough cancellation via lock-in chain	78 ng (SHB30); 781 ng (Nanomag50)	SARS-CoV-2 spike: 125 nM (5 pmol)	Polyclonal anti-spike antibodies	Present work shows substantially lower Fe LOD (1 ng vs 78–781 ng) and targets solution-phase Brownian relaxation delay using mass-normalized harmonic ratios (R_{53}). In present work monoclonal antibodies are used.
**One-step, wash-free SARS-CoV-2 MPS assay** (SI) [3]	One-step wash-free clustering immunoassay	Minimum detectable IPG30 and dilution feasibility for SARS-CoV-2 assays	IPG30 detectability: 265 ng (≈9 fmol nanoparticles)	Not reported in SI	Polyclonal antibodies	Present work employs monoclonal anti-NP, ACE2 protein, and aptamer probes, achieving 5 nM target detection in a controlled two-stage format.
**Wu et al., Nanotechnology, 2019** (review) [4]	Review of MNP-based assays (incl. MPS)	Summary of multiple MPS assay strategies	Not reported	Newcastle disease virus: 150 pM (0.075 pmol); streptavidin: 4 nM (2 pmol); thrombin: 100 pM (0.05 pmol)	Not specified	Present work contributes multi-biomarker experimental validation with explicit ng-Fe hardware sensitivity and harmonic-ratio-based detection
**Wu et al., ACS Appl. Mater. Interfaces, 2020** (H1N1 NP) [5]	MPS with MNP self-assembly / cross-linking immunoassay	Harmonic ratio R_{35} as MNP-quantity-independent metric	Nanogram quantities of iron within 10 s (exact value not reported)	H1N1 nucleoprotein: 44 nM (4.4 pmol)	Polyclonal IgG antibodies	Present work detects lower target concentrations (5 nM, secondary stage) and reports explicit Fe LODs (1–3 ng) using gradiometer-assisted MPS without clustering
**Toward rapid and sensitive detection of SARS-CoV-2…**, ACS Sensors, 2021 [6]	Functionalized MNPs with MPS ratio readout (mimic virus)	Harmonic ratio ΔR_{3}rd/$_{1}$st; imaging-capable system	Not reported	≈0.084 nM (5.9 fmol); ~36 s total measurement	Anti-spike antibodies (type not specified)	Lower concentration LOD achieved using mimic virus model and different workflow; present work provides explicit ng-Fe system sensitivity and multi-target two-stage detection
**This work**	Two-stage detection: probe conjugation followed by target recognition	Gradiometric receive coil with phase/amplitude compensation and signal integration; harmonic attenuation+R_{53}	1 ng Fe (Synomag®); 3 ng Fe (SHP-25)	Primary: 1 nM (ACE2, anti-NP), 12 nM (AS1411); Secondary: 5 nM across all targets	Monoclonal anti-NP; ACE2 protein; DNA aptamer	—

Notes

- NR: not reported in the cited reference
- Fe LOD values refer to total iron mass contributing to the MPS signal
- Harmonic ratios are defined as reported in the respective references

Table S5: Qualitative comparison of the present MPS platform with established diagnostic methods

Method	Main detection principle	Typical strengths	Main limitations	Sample preparation burden	Readout time	Suitability for your study context	Position relative to your MPS system
**Present MPS system**	Magnetic readout of binding-induced Brownian relaxation changes in functionalized MNPs	Fast magnetic readout, simple instrumentation workflow after probe preparation, nanogram-level Fe sensitivity, multi-biomarker compatibility, supports protein-protein / antibody-antigen / aptamer-protein detection	Requires conjugated MNP probes; current study uses controlled iron normalization; full clinical-matrix calibration still under development	Moderate	Short (Fast)	Well suited for rapid relaxation-based biosensing in a controlled assay format	Provides direct magnetic biosensing with simple readout and broad assay flexibility
**ELISA** [7]	Enzyme-linked optical absorbance/fluorescence from antigen–antibody binding	Well established, high specificity, widely accepted in clinical and research labs, strong quantitative protein analysis	Usually requires multiple incubation and washing steps; labeling/enzyme reagents required; workflow can be time-consuming	High	Moderate to long	Strong reference method for protein biomarker validation	ELISA is more established clinically, but our MPS workflow can be simpler and faster under the tested assay conditions
**PCR / RT-PCR / qPCR** [8]	Amplification and detection of nucleic acids	Extremely high sensitivity, excellent for nucleic-acid targets, well standardized	Best suited to DNA/RNA rather than direct protein binding; requires extraction/amplification workflow, specialized reagents and contamination control	High	Moderate	Strong comparator for molecular sensitivity, but not a direct mechanistic match for protein-relaxation MPS assays	PCR is generally stronger for nucleic-acid sensitivity, while our MPS system targets rapid magnetic detection of binding-induced physical changes
**Flow cytometry** [9]	Optical detection of labeled cells or particles in flow	Multiparametric analysis, single-cell capability, excellent for cell phenotyping and population discrimination	Usually requires labeling, more complex sample handling and instrument setup, and is less naturally matched to simple soluble binding readout	High	Moderate	Very useful for cell-based or particle-labeling assays	Flow cytometry is stronger for multiparameter cellular analysis, whereas our MPS system offers a simpler magnetic readout for nanoparticle-based biosensing

Note: The comparison is qualitative because assay sensitivity, specificity, and total workflow time depend strongly on the target biomolecule, labeling strategy, sample matrix, and instrument configuration.

Table S6: Frequency-response measurements of Synomag and SHP-25 samples containing 1 μg Fe

For Synomag: Excitation Frequencies (kHz)			For SHP-25: Excitation Frequencies (kHz)		
**Nominal excitation frequency**	**Measured excitation frequency**	**3rd harmonic Frequency**	**Nominal excitation frequency**	**Measured excitation frequency**	**3rd hamonic Frequency**
1	1	3	1	1	3
5	4.872	14.616	2	1.935	5.805
10	9.744	29.232	5	4.8375	14.5125
15	14.616	43.848	8	7.74	23.22
20	19.488	58.464	11	10.6425	31.9275
25	24.36	73.08	14	13.545	40.635
30	29.232	87.696	17	16.4475	49.3425
35	34.104	102.312	20	19.35	58.05

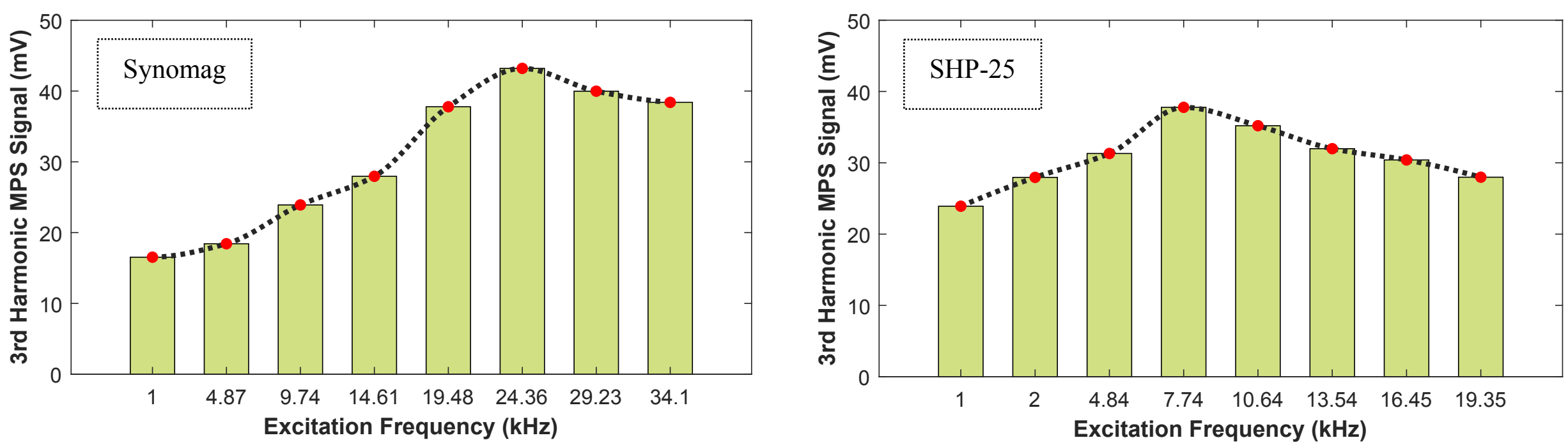

Figure S3. Frequency dependence of the 3rd-harmonic MPS signal for Synomag and SHP-25 samples containing 1 μg Fe in the developed MPS system. The plotted values represent the mean of three measurements from three samples at each frequency, with a measurement time of 10 s per sample. Synomag showed the maximum response at 24.36 kHz, whereas SHP-25 showed the maximum response at 7.74 kHz; these frequencies were therefore selected for the respective sensitivity measurements.

References:

[1] X. Zhang, D. B. Reeves, I. M. Perreard, W. C. Kett, K. E. Griswold, B. Gimi, and J. B. Weaver, "Molecular sensing with magnetic nanoparticles using magnetic spectroscopy of nanoparticle Brownian motion," *Biosensors and Bioelectronics,* vol. 50, pp. 441-446, 2013.

[2] V. K. Chugh, K. Wu, V. D. Krishna, A. Di Girolamo, R. P. Bloom, Y. A. Wang, R. Saha, S. Liang, M. C. Cheeran, and J.-P. Wang, "Magnetic particle spectroscopy with one-stage lock-in implementation for magnetic bioassays with improved sensitivities," *The Journal of Physical Chemistry C,* vol. 125, no. 31, pp. 17221-17231, 2021.

[3] K. Wu, V. K. Chugh, V. D. Krishna, A. di Girolamo, Y. A. Wang, R. Saha, S. Liang, M. C. Cheeran, and J.-P. Wang, "One-step, wash-free, nanoparticle clustering-based magnetic particle spectroscopy bioassay method for detection of SARS-CoV-2 spike and nucleocapsid proteins in the liquid phase," *ACS applied materials & interfaces,* vol. 13, no. 37, pp. 44136-44146, 2021.

[4] K. Wu, D. Su, J. Liu, R. Saha, and J.-P. Wang, "Magnetic nanoparticles in nanomedicine: a review of recent advances," *Nanotechnology,* vol. 30, no. 50, pp. 502003, 2019.

[5] K. Wu, J. Liu, R. Saha, D. Su, V. D. Krishna, M. C.-J. Cheeran, and J.-P. Wang, "Magnetic particle spectroscopy for detection of influenza A virus subtype H1N1," ACS applied materials & interfaces, vol. 12, no. 12, pp. 13686-13697, 2020.

[6] J. Zhong, E. L. Rosch, T. Viereck, M. Schilling, and F. Ludwig, "Toward rapid and sensitive detection of SARS-CoV-2 with functionalized magnetic nanoparticles," *ACS sensors,* vol. 6, no. 3, pp. 976-984, 2021.

[7] Aydin, S.; Emre, E.; Ugur, K.; Aydin, M. A.; Sahin, İ.; Cinar, V.; Akbulut, T. An overview of ELISA: a review and update on best laboratory practices for quantifying peptides and proteins in biological fluids. *Journal of International Medical Research* 2025, *53* (2), 03000605251315913.

[8] Peng, Ruobo, et al. "From PCR to CRISPR: comparing the detection performance of different nucleic acid detection technologies." *Biomedical Instrumentation* (2025): 100026.

[9] McKinnon, K. M. Flow cytometry: an overview. *Current protocols in immunology* 2018, *120* (1), 5.1. 1-5.1. 11.
